# Reviewing the relevance of dioxin and PCB sources for food from animal origin and the need for their inventory, control and management

**DOI:** 10.1186/s12302-018-0166-9

**Published:** 2018-11-01

**Authors:** Roland Weber, Christine Herold, Henner Hollert, Josef Kamphues, Markus Blepp, Karlheinz Ballschmiter

**Affiliations:** 1 POPs Environmental Consulting, Lindenfirststraße 23, 73527 Schwäbisch Gmünd, Germany; 2 Department of Ecosystem Analysis, Institute for Environmental Research, RWTH Aachen University, 52074 Aachen, Germany; 3 Institute of Animal Nutrition, University of Veterinary Medicine Hannover, Foundation, 30559 Hannover, Germany; 4 Öko-Institut e.V, 79100 Freiburg, Germany; 5 Ulm University, 89081 Ulm, Germany

**Keywords:** Dioxin, PCDDs/PCDFs, PCBs, Contaminated sites, Food, Maximum limit, Management measures, POPs, PFOS, PFAS, SCCP

## Abstract

**Background:**

In the past, cases of PCDD/F and PCB contamination exceeding limits in food from animal origin (eggs, meat or milk) were mainly caused by industrially produced feed. But in the last decade, exceedances of EU limit values were discovered more frequently for PCDD/Fs or dioxin-like(dl)-PCBs from free range chicken, sheep, and beef, often in the absence of any known contamination source.

**Results:**

The German Environment Agency initiated a project to elucidate the entry of PCBs and PCDD/Fs in food related to environmental contamination. This paper summarizes the most important findings. Food products from farm animals sensitive to dioxin/PCB exposure—suckling calves and laying hens housed outdoor—can exceed EU maximum levels at soil concentrations that have previously been considered as safe. Maximum permitted levels can already be exceeded in beef/veal when soil is contaminated around 5 ng PCB-TEQ/kg dry matter (dm). For eggs/broiler, this can occur at a concentration of PCDD/Fs in soil below 5 ng PCDD/F–PCB-TEQ/kg dm. Egg consumers—especially young children—can easily exceed health-based guidance values (TDI). The soil–chicken egg exposure pathway is probably the most sensitive route for human exposure to both dl-PCBs and PCDD/Fs from soil and needs to be considered for soil guidelines. The study also found that calves from suckler cow herds are most prone to the impacts of dl-PCB contamination due to the excretion/accumulation via milk. PCB (and PCDD/F) intake for free-range cattle stems from feed and soil. Daily dl-PCB intake for suckler cow herds must in average be less than 2 ng PCB-TEQ/day. This translates to a maximum concentration in grass of 0.2 ng PCB-TEQ/kg dm which is less than 1/6 of the current EU maximum permitted level. This review compiles sources for PCDD/Fs and PCBs relevant to environmental contamination in respect to food safety. It also includes considerations on assessment of emerging POPs.

**Conclusions:**

The major sources of PCDD/F and dl-PCB contamination of food of animal origin in Germany are (1) soils contaminated from past PCB and PCDD/F releases; (2) PCBs emitted from buildings and constructions; (3) PCBs present at farms. Impacted areas need to be assessed with respect to potential contamination of food-producing animals. Livestock management techniques can reduce exposure to PCDD/Fs and PCBs. Further research and regulatory action are needed to overcome gaps. Control and reduction measures are recommended for emission sources and new listed and emerging POPs to ensure food safety.

## Background

Polychlorinated dibenzo-*p*-dioxins, polychlorinated dibenzofurans (PCDD/Fs), and polychlorinated biphenyls (PCBs) are widely recognized environmental and food contaminants [1, 2]. The use of PCBs and their weak life-cycle management have resulted in a widespread contamination of the technosphere and the environment. Former open applications such as sealants are still contributing to environmental release and human exposure [3]. Similarly, the release of PCDD/Fs over the last two centuries’ industrial emissions and one-century chlorine/organochlorine production has impacted and contaminated soils and sediments, generating contaminated sites and hot spots [4–9].

Humans are exposed to dioxins and PCBs mainly via food, especially through consumption of animal-derived foods such as meat, dairy and eggs, and fishery products (BMU) [10]. Fruits, vegetables, nuts and cereals have normally low levels of PCDD/PCDFs and PCBs [11], but due to high consumption they also contribute to the food-borne uptake of these pollutants [12].

In 2002, the EU set maximum levels for PCDD/Fs in certain foodstuffs, and set maximum levels for the sum of PCDD/Fs and dioxin-like PCBs (dl-PCBs) in 2006 [13]. The regulation was amended in 2011, introducing new EU maximum levels for PCDD/Fs and the sum of PCDD/Fs and dl-PCBs, based on World Health Organization (WHO) toxicity equivalency factors derived in 2005 (TEF 2005), and establishing maximum levels for non-dioxin-like PCBs (ndl-PCBs) [14].

Average PCDD/F and PCB levels have decreased in many countries compared to levels in the 1990s, which is also reflected in decreasing PCB and PCDD/F levels in human milk [15]. Most of the meat and milk samples on the European market meet the regulatory limits (EFSA) [16].

However, in the past 10 years, PCB contamination of meat and eggs has been detected more frequently [17], following the inclusion of dl-PCBs in the EU regulation in 2006.

Animal feeds and feed additives are major sources of dioxin and PCB contamination for food of animal origin. Feed incidents have traditionally been the main reason for exceeding maximum levels of PCDD/Fs and PCBs in food of animal origin [1], such as the Belgian PCB and dioxin incident in 1999 [18, 19], the citrus pellet case from Brazil [20, 21], the Irish pork scandal [22, 23], the Chile pork contamination [24] and the bio-diesel incident in Germany [25], which mainly impacted large agroindustry farms [2, 26]).

In recent years, sheep (in particular sheep liver) and beef [27–29] from free-range production have also exceeded the EU maximum limits for the sum of PCDD/Fs and dl-PCBs, even without specific contamination of feedstuffs. Eggs from laying hens housed outdoors are particularly sensitive indicators of PCDD/F and PCB contamination in soil and can also be a relevant exposure pathway for humans [17, 30]. This has increasingly been recognized in the last decade, resulting in a growing number of reports of PCDD/F and PCB contamination of eggs [30–34]. The contemporary relevance of this contamination has recently been demonstrated in a monitoring study, which found that more than 50% of the eggs from 60 small flocks in the Netherlands exceeded the EU maximum limit [31].

In a study initiated by the German Environment Agency we assessed the impact of environmental PCDD/F and PCB contamination on products of animal origin, and evaluated the significance of various sources [17]. The study surveyed contaminated soils and feedstuffs linked to food from animal origin that exceeded EU maximum PCDD/F and PCB levels, and established critical limits for soil contamination. The assessment revealed that free-range broiler/eggs and beef cattle from extensive farming may get contaminated with dioxin-like PCB and PCDD/F even when soils display relatively low levels previously considered as safe. Therefore, more stringent soil standards and emission source control are needed. This paper gives an overview of the key findings, including a compilation of pollution sources identified in surveys and food safety incidents in Germany regarding cattle herds or chicken flocks with PCDD/F or PCB levels above EU maximum limits. It also reviews the literature reporting cases and practical experiences of PCDD/F and PCB exposure sources.

The Food and Agriculture Organization of the United Nations (FAO) and the Intergovernmental Technical Panel on Soils (ITPS) have recently identified soil pollution as one of the ten major soil threats listed in the 2015 Status of the World’s Soil Resources report [35]. Persistent organic pollutants (POPs) including PCDD/Fs and PCBs are among the most relevant soil pollutants [36]. There is an urgent need to eliminate pollution sources and to control, secure and remediate contaminated sites and reservoirs, to reduce exposure and guarantee food safety. This compilation of the major PCDD/F and PCB sources and their potential pathways to contaminated soils and food-producing animals can inform the development of appropriate source monitoring and reduction measures.

## Results and discussion

### Relevance of dietary intake to human exposure

Dietary intake is the predominant exposure pathway to PCDD/Fs and PCBs. For PCDD/Fs, more than 90% of daily intake is due to consumption of food from animal origin. For PCBs, indoor dust and air also constitute a significant risk to sensitive subpopulations [37, 38]. Different organisations have undertaken risk assessments of dioxins and dl-PCBs and developed a range of health-based guidance values (HBGV) [39]. In 1998, the World Health Organization (WHO) proposed a TDI for PCDD/Fs and dl-PCBs of 1–4 pg toxic equivalents (TEQ)/kg body weight (bw) per day [40]. In 2001, the Scientific Committee on Food (SCF) of the EU established a tolerable weekly intake (TWI) of 14 pg TEQ/kg bw per week. This TWI is in line with the provisional tolerable monthly intake (PTMI) of 70 pg TEQ/kg bw per month set by the Joint FAO/WHO Expert Committee on Food Additives (JECFA) in 2001. For total PCBs, the World Health Organization (WHO) derived a tolerable daily intake (TDI) of 20 ng PCB/kg bw per day in 2003 (WHO) [41]. Currently the European Food Safety Authority (EFSA) is reassessing the human health risk related to the presence of PCDD/Fs and dl-PCBs in food. The TWI is being revised and will likely be reduced [42].

A part of the German adult population still exceeds both the current valid TWI of 14 pg TEQ/kg bw for the sum of PCDD/Fs and dl-PCBs, and the tolerable daily intake (TDI) for total PCBs of 20 ng/kg bw [43]. For children, PCB intake is about 2.5-fold, and for breast-fed infants about 50–100 times the adult intake [44], which is similar for PCDD/Fs.

### Exposure of food-producing animals^1^ and accumulation of PCDD/Fs and PCBs

Some types of livestock farming are prone to accumulate PCDD/Fs and PCBs. Kamphues and Schulz [45] categorised food-producing animals according to their exposure risk to PCDD/Fs in soils. Due to similar physico-chemical properties, the environmental exposure to PCBs from soils and vegetation (including feeds like grass and hay) is similar to that of PCDD/Fs. However, the bioaccumulation of PCDD/Fs and PCBs depends on congener, species, and tissue. As a result, bioaccumulation from feed/soil to food of animal origin changes the congener patterns considerably [2]. It is possible to determine congener-specific rates for the transfer (carry-over rates) from soil/feed to livestock products (meat, eggs, milk). For congeners critical to EU maximum limits (i.e., the TEQ-relevant congeners with major contribution from PCB-126 and non-dioxin-like PCB-138, -153, and -180), comparable carry-over rates have already been determined [46, 47].

This project developed exposure assessments for beef cow herds and free-range hens, and further assessed exposures for other food-producing animals, depending on feeding and housing [17].

To elucidate why maximum levels in livestock products are exceeded, it is necessary to know the critical total daily dioxin/PCB intake for each species that makes a specific livestock product (e.g., egg, milk, meat) exceed the EU maximum levels. From this information, it is possible to derive the dioxin and PCB contamination in the soil and feed that leads to non-compliance with the EU maximum level for the respective livestock product.

Depending on the animal (and the exposure pathway and source), PCDD/Fs or dl-PCBs can contribute in various ratios to TEQ. dl-PCBs are often the main contributor. For example, in the German federal monitoring plan for beef from suckler cow herds, more than 90% of the regulatory TEQ exceedances in meat were from dl-PCBs. On the other hand, in 60 investigated small free-range chicken flocks in the Netherlands, the TEQ contribution from PCDD/Fs and dl-PCBs was similar, with a slightly higher impact from PCDD/Fs [31].

#### Free-range chickens and eggs

Free-range chickens are particularly prone to environmental contamination. They take up more soil than other farm animals per body weight. A PCDD/F content in the feed of 0.4 ng TEQ/kg dry mass (dm), which is at about 50% of the EU maximum level for feed (0.75 ng PCDD/F-TEQ/kg 88% dm), is already sufficient to exceed the EU maximum level for PCDD/Fs in eggs [46].

Free-range laying hens and broilers ingest on average about 11 g and up to 30 g soil per day [46, 48]. Some recent studies have shown that dioxin and dl-PCB levels in eggs from free-range chickens frequently exceed EU food standards of 2.5 pg TEQ/g fat for PCDD/Fs or 5 pg TEQ/g fat for the sum of PCDD/Fs and dl-PCBs at soil concentrations around 2–4 ng PCDD/F-TEQ/kg dm [17, 31, 32, 34]. For hens ingesting approx. 30 g soil per day, models indicate that soil levels around 2–4 ng PCB-TEQ/kg can be high enough to reach or exceed the EU standards. This is particularly important for flocks of chickens spending a lot of time outside, given their higher soil intake [32]. The average exposure depends on the size of the chicken flock, because flock size is related to the time chickens spend outside. Flocks with fewer than 500 hens spend 40% or more of their time outdoors, while in farms with more than 10,000 hens the animals are outdoors less than 10% of the time [32]. A second variable is the strength of bound PCDD/Fs. For sources where PCDD/Fs in soils are bound to, e.g., activated carbon, the extractability is lower and, therefore, the problematic levels would be higher.

**Fig. 1:**
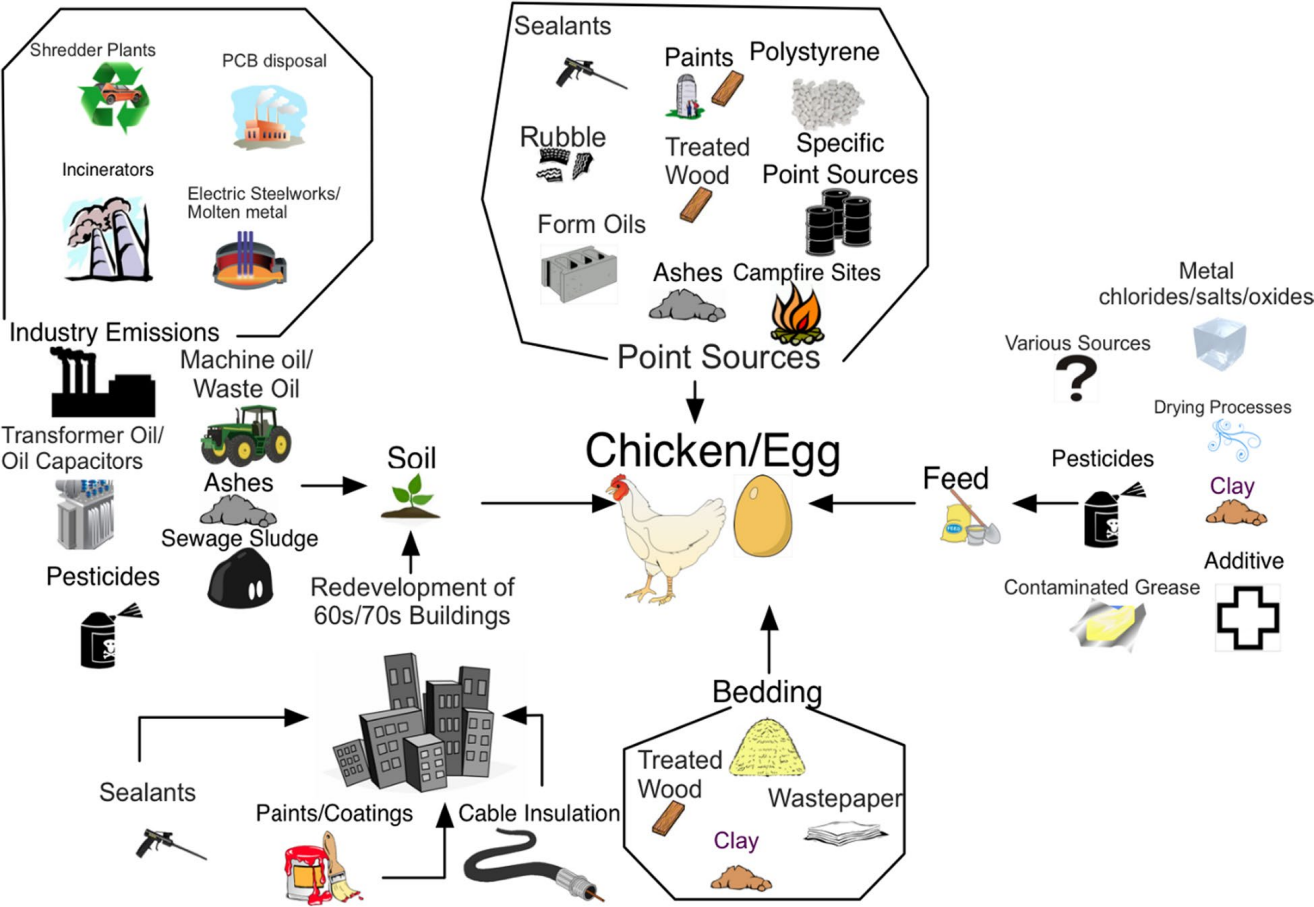
PCB and PCDD/F exposure sources for chicken/egg

Therefore, soil—chicken egg is probably the most sensitive exposure pathway for PCBs and PCDD/Fs from soil to humans. People—and especially young children—consuming contaminated eggs can easily exceed health-based standards and may be subject to high exposure levels. With the consumption of a single hen’s egg (avg. 7 g fat) per day, a 4–5-year-old child (weighing 16 kg) would exceed the TDI of 2 pg TEQ/kg bw, even if the egg complied with the EU regulatory limit for eggs of 5 pg PCDD/F–PCB-TEQ/g fat. The current regulatory limit for soil for residential areas and private estates in Germany or Netherlands, for example, is 1000 ng PCDD/F-TEQ/kg dm. Eggs from chickens kept on land with these levels of contamination could contain approx. 800 pg TEQ/g fat. In this case, a single egg would exceed the TDI for a 16-kg child by approximately 175 times. Therefore, it is clear that consuming contaminated eggs can easily lead to exceeding health-based standards—especially for young children. Levels of > 5 ng TEQ/kg dm would certainly be too high and would require either that production is stopped or access to free-range areas is restricted. Therefore, it has been suggested that soil used for the production of free-range eggs should ideally contain less than 2 ng TEQ/kg dm both for PCDD/Fs and for dl-PCBs. These levels are below all current national soil standards but are critical for the safe production of free-range eggs and chicken meat. Other pathways also need to be considered when assessing PCB and PCDD/F exposure sources, including chicken feed and bedding in the henhouse (Fig. 1).

#### Cattle: in particular beef and veal from suckler cow herds

**Fig. 2:**
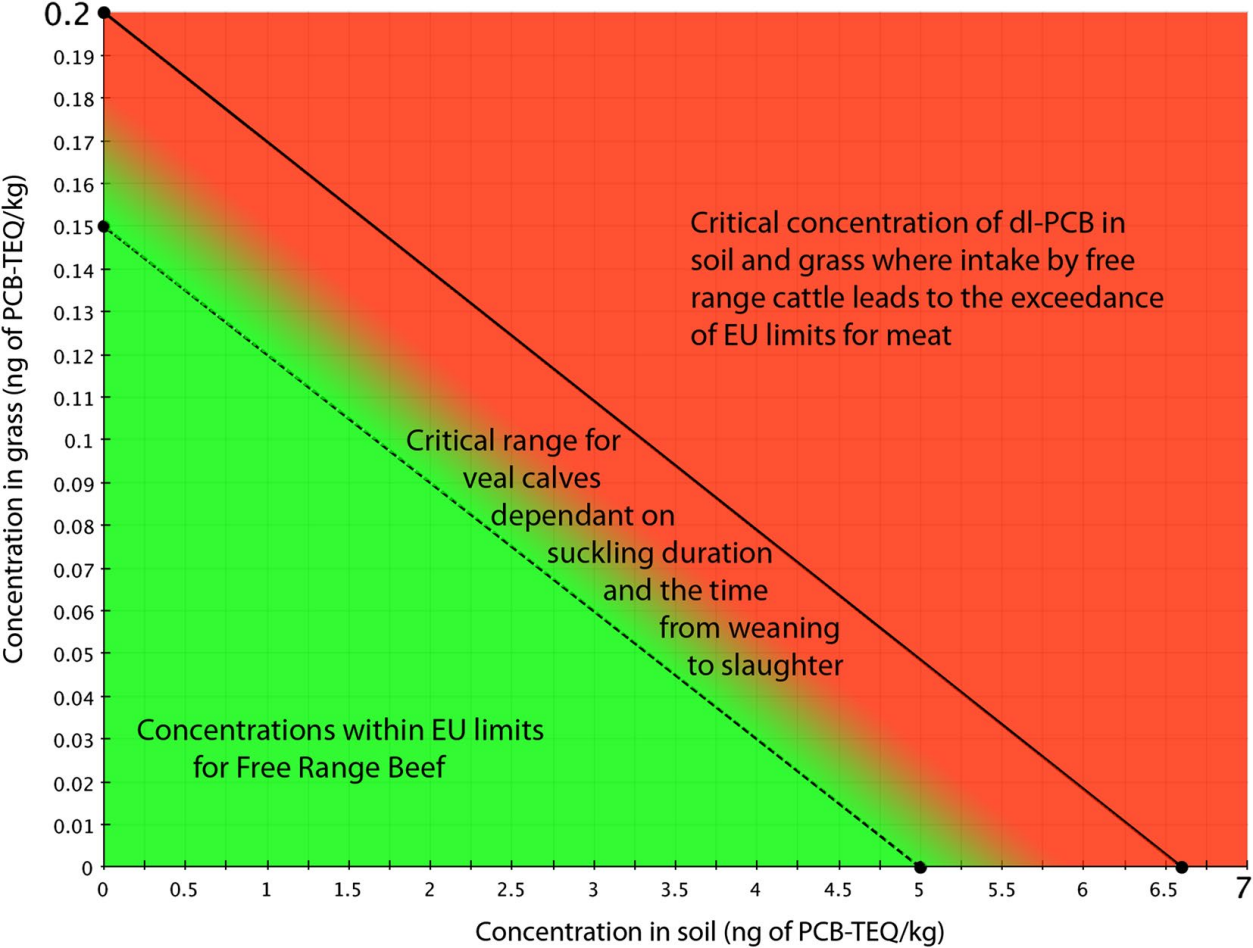
Cumulative impact of grass and soil contaminated with dl-PCBs on exceedance of EU maximum levels for the sum of dioxins and dl-PCBs in beef and veal from suckling calves. Concentrations above the solid line are critical for beef and concentrations above the dashed line are critical for veal from suckling calves [17]

Cattle are particularly sensitive to exposure from environmental PCB contamination [17, 49]. Cattle take up PCBs and PCDD/Fs from feed, including from feed contaminated with soil particles (e.g., grass, grass silage or hay). Grazing cattle are also exposed to contaminated soil during grazing. The amount of soil intake depends on the quality of the meadow and amount of grass available. For a high-yielding meadow, soil constitutes a minimum of 3% of the ingested grass mass [10, 50]. A total daily intake of a beef cow of approx. 2 ng PCB-TEQ/day from both feed and soil might be sufficient for exceeding the maximum/upper limits for beef and veal (Fig. 2; [51]), particularly when calves are allowed to suckle their mother’s milk for several months to a year. Considering an average consumption of 10 kg dm of grass/hay with a minimum soil content of approx. 3%, meat from these suckling calves can exceed the EU regulatory limits at relatively low soil levels, below 5 ng PCB-TEQ/kg dm (Fig. 2), combined with grass/feed levels around 0.15 ng PCB-TEQ/kg dm. These critical grass levels are considerably below the EU regulatory limits for feed of plant origin of 1.25 ng TEQ/kg (moisture content of 12%) for the sum of PCDD/Fs and dl-PCBs [3, 17].

This was confirmed in practice with herds with dl-PCB levels that exceed TEQ limits for meat, where no point sources could be identified. Thus, relatively low soil levels (below 5 ng PCB-TEQ/kg dm) in combination with feed levels around 0.15 ng PCB-TEQ/kg dm seem to be responsible for non-compliance with EU limits in meat.

**Fig. 3:**
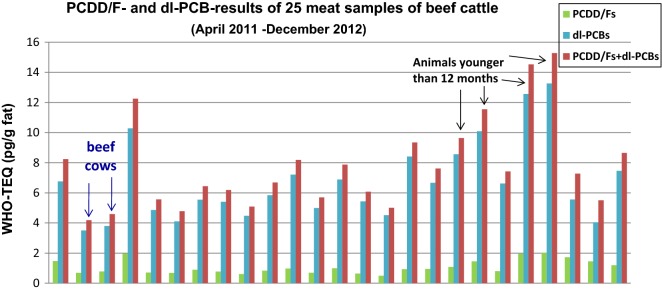
PCDD/F and dl-PCB TEQ levels in meat of beef cattle on a pasture with elevated PCB–soil levels (mean 2 ng WHO-PCB-TEQ/kg dm, range 0.7–5.6) [29]

**Fig. 4:**
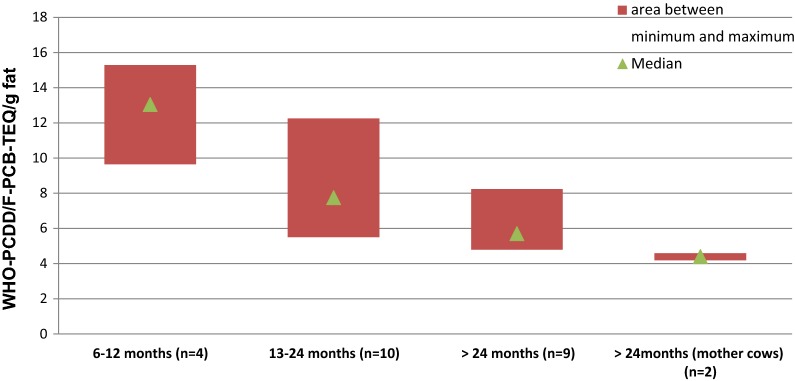
TEQ levels in meat of cattle within one suckler cowherd in relation to age at slaughter [29, 52]

25 beef samples from one herd that exceeded the EU regulatory limit for meat were analyzed for PCDD/Fs and dl-PCBs ([52]; Fig. 3). dl-PCBs were the main TEQ contributor (86% of total TEQ on average). The herd was grazing on a former military area with soil levels up to 5.6 ng PCB-TEQ/kg dm. Meat samples from beef cows (number of samples: 2) had PCDD/F–PCB-TEQ levels around the EU maximum level of 4 pg TEQ/g fat, whereas most samples from calves and other beef cattle clearly exceeded the maximum level (Fig. 3). Figure 4 shows the PCDD/F–PCB-TEQ levels in meat according to the age of the slaughtered animal. Samples from suckled calves (age 6–12 months) had about two–three times the levels of beef cattle after weaning and feeding on grass for several months (Fig. 4). This suggests PCDD/F and PCB levels in beef cows are reduced by lactation, transferring these contaminants to the calf (similar in humans).

#### Sheep/goat

For sheep and goat, in principle, the same applies as for beef cattle production. The critical levels in feed [53] and soil are of the same order of magnitude as for suckler cow herds.

Sheep are considered among the most sensitive animals [27]. Grazing sheep nip closer to ground surface, and the share of ingested soil may be high, up to 20% of forages. For dairy sheep, Brambilla et al. [54] computed the carry-over of PCDD/Fs and dl-PCBs from soil to milk for different soil intake scenarios. They found that for high soil intake, sheep milk may not comply with the EU maximum limit for milk (5.5 pg PCDD/F–PCB-TEQ/g fat) when the contamination of soil is above 4 ng PCDD/F–PCB-TEQ/kg dm [54].

Sheep may be exposed to more highly contaminated soils because they often graze on areas near rivers or industrial sites, along streets, or on former industrial or military areas. However, in a German survey [55] the percentage of sheep meat above EU maximum levels was lower than that of beef from beef cow herds, and critical PCB/PCDD/F levels in the soil and grass/feed seem to be slightly higher for sheep than for the offspring of beef cows. One possible reason for the lower levels in sheep meat might be the high accumulation of PCDD/Fs and some dl-PCB congeners in sheep liver. Prior to the amendment of the EU maximum levels for liver of terrestrial animals in 2013, over 90% of sheep livers in Germany exceeded the EU maximum levels [55], and the Federal Institute for Risk Assessment (BfR) warned against consumption [56]. After the amendment of the EU maximum limit, only approx. 10% of German sheep livers exceed the EU limit.

To protect human health, it is not sufficient to be below EU maximum levels for dioxins and dl-PCBs, human consumption must also comply with the TWI of 14 pg TEQ/kg bw. For children in southern Italy, 38% of the dietary exposure to dioxins and dl-PCBs is via dairy products. For high soil intake of sheep, Brambilla et al. [54] calculated that the TEQ concentration in soil has to be as low as 0.74 ng TEQ/kg dm to ensure protection of children’s health.

PCBs and PCDD/Fs have also been detected in goat products (milk, meat) due to environmental contamination [57, 58].

#### Pigs

Kamphues and Schulz [45] listed wild boars and pigs housed outdoors in the highest PCDD/F exposure category of food-producing animals, together with chicken [45]. Because of their feeding behavior, pigs housed outdoors and wild boars are at high risk of PCDD/F exposure via soil, as they find a large part of their feed on and in the soil. However, while there are data showing elevated PCDD/F and PCB levels in wild boar (see below), there are no reported cases of pigs housed outdoors exceeding regulatory limits for PCDD/Fs or PCBs. On the other hand, cases of pork meat with PCDD/Fs and/or dl-PCBs exceeding the EU maximum levels are known from industrial pork contamination scandals due to industrial feeds produced using contaminated materials or ingredients [18, 22–25]. In a recent case of PCB contamination in pigs, the source was not the soil but an old tank on the farm painted with PCB paint flaking off into the area where the pigs were housed [59]. Soil PCDD/F or PCB levels that may lead to exceedances of EU limits in pork meat have not been derived yet.

#### Game

It is well known that deer, like sheep and goats, eat the grasses and plants very close to the soil, resulting in increased uptake of soil particles. Therefore, for deer—similar to cattle and sheep—the soil next to the feed can serve as a relevant PCDD/F and PCB exposure pathway. In addition, forest soils, especially in populated areas, have a higher PCDD/F and PCB content than grasslands due to the adsorption of pollutants via leaves and transfer to the topsoil [60]. PCDD/F and dl-PCB levels in liver of deer and wild boar in central Europe are generally high and are elevated in some meat [17, 61, 62]. In Germany the livers of deer (median 45.2 pg PCDD/F–PCB-TEQ/g fat) and wild boar (median 50.8 pg PCDD/F–PCB-TEQ/g fat) showed very high levels, well over the EU maximum level of 12 pg TEQ/g fat applicable to bovine or ovine livers up to 2011. The EFSA Panel on Contaminants in the Food Chain (CONTAM Panel) concluded that “frequent consumption of deer liver, especially for high consumers may be of health concern” and that “frequent consumption of sheep liver, particularly by women of child-bearing age and children, may be a potential health concern” [27]. For wildlife, however, there are no regulatory PCDD/F or dl-PCB limits in the EU.

Critical PCDD/F or PCB levels in soils or vegetation have not been derived for game and cannot be related to regulatory limits, which are absent for wild animals. However, limits exist for meat from deer breeding.

### Background levels in German soils and pasture grass

The German EPA [63] analyzed 500 background soil samples in Germany. The dl-PCB levels in grassland topsoils were a factor of 10–20 below the critical dl-PCB level of approx. 5 ng PCB-TEQ/kg dm for free-range beef, and a factor 5–10 below critical levels (2–4 ng dl-PCB-TEQ/kg dm) for free-range hen eggs.

PCDD/F levels in background farmland soils are higher, with a median of 0.5–1.1 ng TEQ/kg dm depending on the carbon content [63]. Nevertheless, they are a factor of 3–8 below critical levels (2.5–5 ng PCDD/F-TEQ/kg dm) needed to contaminate eggs above EU regulatory limits.

dl-PCB background levels in grass in German rural areas (median 0.06 ng PCB-TEQ/kg 88% dm) are on average a factor of 2–3 below the critical dl-PCB levels for beef cow herds of 0.15–0.2 ng PCB-TEQ/kg 88% dm (Table 1). The dl-PCB (and PCDD/F) concentrations in grass increase with population density. In more populated areas (median 0.13 ng PCB-TEQ/kg 88% dm) they approach critical levels, while in a city like Munich the dl-PCB levels are around 0.4 ng PCB/kg dm (see below; [64]).

**Table 1:** Levels of PCDD/Fs and dl-PCBs in feed samples (grass, hay, grass silage), results of the monitoring programme in the German State of Baden-Württemberg

Feed/grass Location of sampling	PCDD/Fs (1996–2012)		dl-PCBs (2005–2012)	
	Range	Median	Range	Median
	(ng WHO (2005)-PCDD/F-TEQ/kg 88% dm)		(ng WHO (2005)-PCB-TEQ/kg 88% dm)	
Rural area	0.03–0.26	0.06	0.05–0.18	0.07
Rural area	0.02–0.31	0.08	0.04–0.21	0.06
Slightly populated area	0.03–0.33	0.09	0.05–0.18	0.10
Populated area	0.03–1.04	0.12	0.07–0.47	0.13
Populated area	0.02–0.42	0.11	0.07–0.37	0.13

Thus, levels of PCDD/F or dl-PCB in background soils or feed in Germany (a country with high use of PCBs in open application) are below critical levels even for exposure-sensitive animals (chicken/egg or beef cow herds). Therefore, specific PCB or PCDD/F contamination of soil and/or feed is needed to contaminate food from animal origin above regulatory limits. In densely populated areas in West Germany the PCB levels in grass already contribute a considerable share of the problematic PCB intake for beef via feed.

The following sections summarize PCB and PCDD/F contamination sources and the levels found in the German survey, and briefly describe the experience of German federal state governments. Additionally, relevant information from scientific literature and studies in other countries are included, to provide an overview of PCB and PCDD/F contamination sources for food-producing animals. The study does not address specific contamination sources for feed incidents, which have recently been addressed in another publication [1].

### PCB sources with potential relevance to food-producing animals

#### Use of PCBs in Germany and air emissions from open applications

Germany possesses one of the most detailed assessments of past use of PCBs and can, therefore, be used as a case study for the use and management of PCBs. In Germany, 85,000 tonnes of PCBs was marketed in various applications: 72,500 tonnes in West Germany and 12,330 tonnes in East Germany [65, 66]. The amount of PCB-TEQ can be estimated as approx. 425–1000 kg TEQ. This shows the potential for environmental and food contamination with dl-PCBs, when compared, for instance, with the current release rate of PCDD/Fs of approx. 68 g TEQ/year in Germany and approx. 100 kg TEQ for the yearly entire world emission today [67].

In West Germany, 48,000 t of PCBs was used in closed applications mainly in capacitors (13,000 t), transformers and other electrical equipment (23,000 t), and hydraulic fluids (12,500 t) [65]. 24,000 t of PCBs produced by Bayer (Clophen mixtures) was used not only in open applications, in sealants/caulking, but also in paints/varnishes, cable sheaths, adhesives, and other products, and as lubricating oil [65]. Of this amount, 20,000 t was used as plasticizer in sealants in the construction sector by the Thiokol company [68]. Since Thiokol had also imported Aroclor from Monsanto for sealant production, the total PCB amount in open applications must be even higher than 24,000 t [68].

In the former East Germany, about 11,000 t of PCBs was used in capacitors. 1000 t of PCBs was added as plasticizers to PVC paints and PVC sheathing of power cables in open application [65].

Considering the estimated stock of 12,000–19,000 t of PCBs in buildings and constructions in West Germany and an annual evaporation rate of 0.06%, this results in an estimated 7–12 t of PCB per year emitted from the remaining PCBs in open applications [17]. Other PCB emission estimates, for instance in Switzerland, are also in agreement: using air measurements, Bogdal et al. [69] calculated PCB emissions for Switzerland of approx. 1.5 t per year. Recalculating the population of West Germany, this would correspond to an annual PCB emission of approx. 11.4 t. The per capita consumption of PCBs in open applications in West Germany (375 g/person) was one-third higher than in Switzerland (280 g/person) [70]. Therefore, the actual West German PCB emissions is expected to be slightly higher, approx. 15 t, and in the order of magnitude of the estimated desorbed 7–12 t PCB per year and far higher than the official German PCB inventory of 220 kg [71].

Furthermore, PCB-containing buildings and other constructions (e.g., bridges, swimming pools, pylons, pipelines, dams, ships) from the 1950s to 1970s increasingly need repair and maintenance work. If maintenance, repair or demolition measures are not carried out in a professional manner, larger quantities of PCBs are released uncontrolled into the environment within a few days than in the previous decades by desorption. In particular, when removing PCB-containing paint and anticorrosion coating or cleaning facades with joint sealants by abrasive blasting, large amounts of PCBs can be released and contaminate soils, sediments, water bodies and plants [70, 72–77]. This release might even be larger than the annual 7–12 tonnes of desorbed PCBs [76].

The German PCB inventory (release of 220 kg/year) only considers the emission of unintentionally formed PCBs from combustion plants and other thermal sources [71] neglecting the PCB release from remaining open application. Environmental matrices (soil, air and grass) contain almost exclusively PCB–congener profiles of industrially produced PCBs. This demonstrates the dominance of industrially produced PCBs emission sources and the low relevance of thermally unintentionally formed PCBs, and supports the emission estimate.

Overall, PCB emission and contamination of the environment, including vegetation, e.g., grass or spruce needles, have decreased in the last 20 years in Germany [64].

The sources of PCBs for food-producing animals can be divided into local sources at the farm or pasture and regional sources impacting larger areas (also impacting the pasture areas). The PCB sources impact soil to varying degrees as exposure pathways (in particular, historic contaminations). Contemporary PCB emission sources impact grass/feed via atmospheric deposition.

**Fig. 5:**
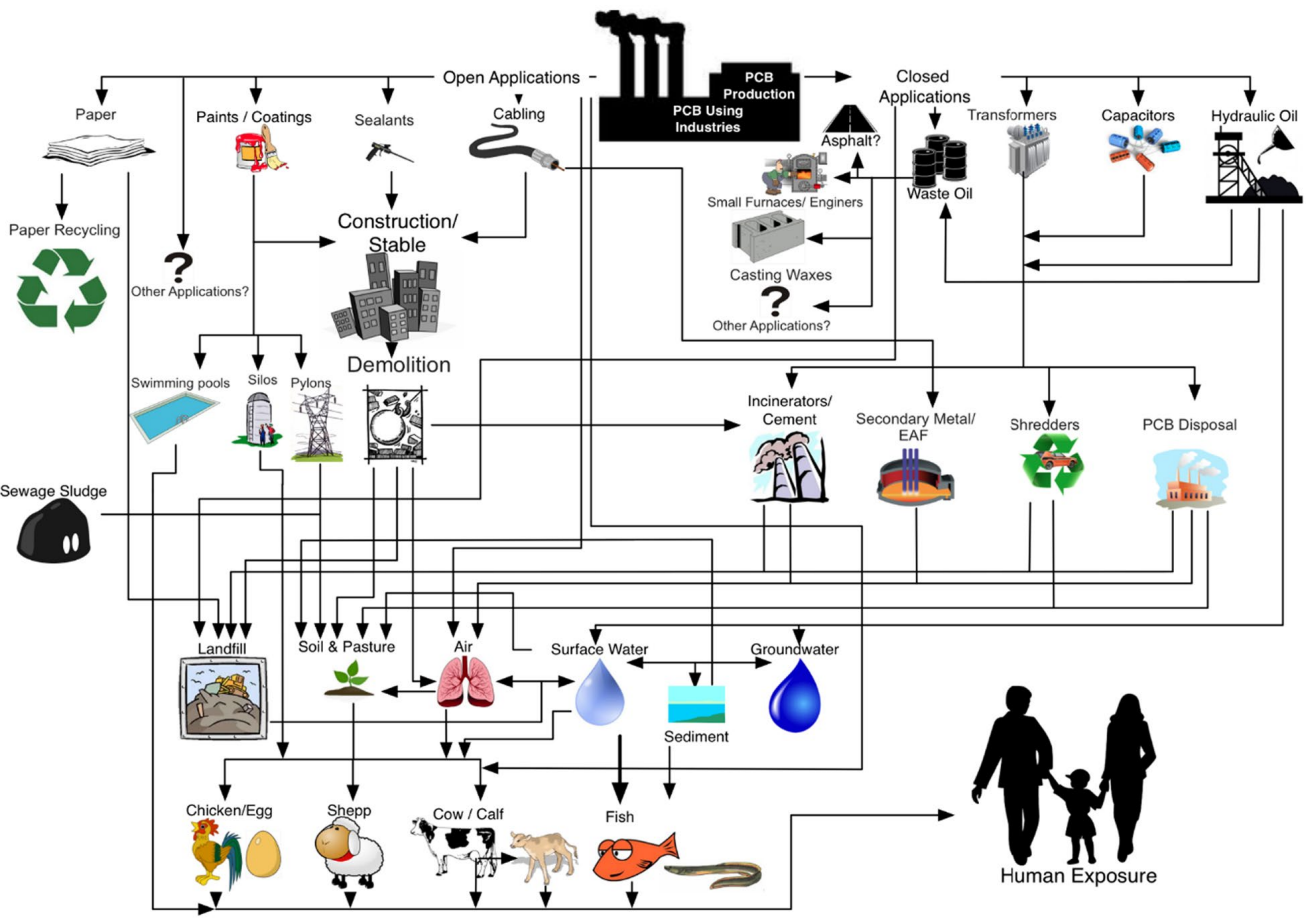
Life cycle of PCBs showing environmental release sources and exposure pathways for food-producing animals [17]

Figure 5 illustrates the life cycle of PCBs [PCB production–use in production (e.g., production of paints or transformers)–product use–recycling–end of life] and gives information for identifying PCB sources and potential entry routes of PCBs into animal products for the individual stages of the PCB life cycle (Fig. 5), which is detailed in the following paragraphs.

#### PCB production sites and related contamination and exposure

Animals and humans have been exposed to PCBs around production sites. The contamination and exposure via animal products have been documented for the PCB production sites in Anniston/US [78] and Brescia/Italy [79]. At the former PCB production site in Michalovce/Slovakia, high environmental contamination [80] and elevated human exposure were found up to 70 km in wind direction of the plant, with food animals as likely major human exposure pathways [81].

For some sites, no information on contamination is available at all. At the East German PCB production site (Deutsche Solvay Werke, Westeregeln), the factory was destroyed by a major fire on 15 January 1961 [82, 83]. The PCB and PCDF releases from this fire could have been substantial and contamination might be relevant, but no assessment has been reported. Also for the large production of the Bayer Company in Leverkusen in West Germany no documentation of associated impact has been published.

#### Industries having used PCBs and related contamination

Some manufacturing industries used PCBs for the production of transformers, capacitors, paints, sealants, flooring, paper or textiles. Such companies have used PCBs in the 100–1000 tonne range, with associated releases and contamination. In two factories in East Germany, manufacturing capacitors containing PCBs, approx. 580 t of PCBs from the 11,243 t used in production has been lost during handling [65]. A company producing PCB-containing carbonless copy paper has released more than 50 t of PCBs into the environment [84]. These released or disposed tonnes of PCBs at such sites impact the surrounding.

Hen eggs sampled from two private chicken holders (Teningen/southwest Germany) close to a capacitor factory producing PCB containing capacitors for some time were highly contaminated with dl-PCBs. PCB levels in eggs were approx. 25 pg PCB-TEQ/g fat at both locations and thus five times above regulatory limits. Soil levels in these areas were six–ten times above German background levels of approx. 0.5 ng PCB-TEQ/kg dm [63]. Also, fish were analyzed in the receiving water from the capacitor company, which was used by the local fishing association. One eel sampled contained a high dl-PCB level of 259 pg TEQ/g fresh weight [85]. With a 200 g portion of the eel, an adult would ingest a PCB-TEQ dose equal to the tolerable intake for 1 year. For a child (16 kg), a 100-g portion contains as much TEQ equal to a tolerable intake for 2.2 years. Despite the known contamination potential of the factory and the consumption of fish from the direct receiving waters, no assessment of food-producing animals has been conducted for more than 30 years.

In Switzerland, another capacitor factory disposed capacitors to a landfill. The capacitors corroded, released PCBs over decades, and contaminated a river and its fish up to a distance of approx. 40 km downstream of the landfill [86, 87].

These cases highlight the need for monitoring current pollution from factories that used tonnes of PCBs in the past, as well as determination of contaminant levels in animal-derived foods in the surrounding area.

#### Industries having used PCB-containing equipment and related contamination

A range of industries have used PCB-containing equipment or PCB oils. This includes, for example, companies operating the electricity grid and high-energy-consuming industries such as steel/metal production and power plants. Contamination can occur where transformers are operated or receive maintenance and where old transformers, equipment with hydraulic fluid and other PCB-containing equipment are stored.

The inventory of a primary steel plant in Austria revealed that 106 t of PCBs has been used in transformers and capacitors at this location [88]. At another large steel plant in Italy, 1000 transformers were in operation. Such PCB containing transformers have an estimated average yearly leakage rate of 0.06–0.3 kg/t [9, 89]. Around the Italian steel plant, PCB (and PCDD/F) contamination of cattle (meat, milk) above EU regulatory limits had been discovered, which led to 1600 sheep/goats needing to be destroyed. The PCB soil contamination (average 3.3 ng PCB-TEQ/kg dm within the 5 km zone; maximum 25 ng PCB-TEQ/kg dm) [90–92] stems mainly from the historic fugitive PCB emissions of the PCB-containing equipment on-site [93]. Grazing cattle are now banned on a radius of 20 km around the steel plant [90–93].

Also, industries treating, cutting or pressing metals have used PCB-containing metal working fluids such as cutting oils [94]. PCBs have been released from this open application and metal residues containing such oils have most likely ended up in sinter plants that recover metal waste, leading to further environmental releases.

Secondary smelters release PCBs not only from operating equipment but also from metal scrap containing PCB paints and anticorrosion coatings, (small) capacitors or other metal scrap contaminated with PCBs. A secondary metal smelter in Switzerland was responsible for the contamination of a river and its fish [87].

Egg monitoring conducted by the International POPs Elimination Network (IPEN) detected high TEQ levels (dl-PCB and PCDD/F) in eggs around metal industries, with more than 90% of eggs exceeding the EU regulatory limits for food [30, 34, 95].

PCBs have also been used in military applications (hydraulic oils, transformers, paints for equipment/vehicles and sealants) [17, 73, 96–98]. One of the contaminated beef herds in the German monitoring study was grazing on a rehabilitated military area [17, 29]. PCB contamination has also been reported from US/NATO bases [96, 97].

PCBs were also used in large volumes in the mining industry as hydraulic oil. In Germany, from the approx. 12,500 tonnes of PCB used in mining, only 5% has been managed appropriately, and the remainder has been released into the mines [65, 99]. PCBs leak into rivers from pumped water. In the German mining areas in North Rhine-Westphalia and Saarland, high levels of PCBs in fish have been documented, and eel was deemed unfit for human consumption [100, 101]. Beef and sheep on some flood plains in North Rhine-Westphalia exceeded the EU regulation limit [17, 49]. These PCBs in sediments and flood plains originate also from other sources in addition to mining, including large chemical industries [102, 103], metal industries (most likely), and open applications impacting grass and other fodder via atmospheric deposition [17].

#### PCB use in open applications and related contamination

##### Direct exposure to PCBs in stables and on farms

PCBs have been used also on farms in paints, coatings and sealants. Open use of PCBs in fodder silos was a major food contamination pathway of cattle/milk detected in the 1970/80s [99, 104]. However, cases of meat and egg^2^ contamination above EU regulatory limits from open PCB uses on farms were recently discovered in Europe:

- PCB paint in silos, on asbestos roofs and PCBs in a rubber conveyor belt have contaminated chicken/eggs and beef [17, 105, 106].
- PCB paint/coating on a metal surface has contaminated pork meat [59].
- PCB paints on the walls of a stable contaminated a beef herd [107].

This demonstrates that PCBs in open applications, in particular in paints, are still present in stables and in the surrounding area and are of contemporary relevance to food-producing animals.

##### PCB release from open application during construction/maintenance work

PCBs can be released into the environment due to improper handling of PCB-containing materials, during demolition, removal, refurbishment or maintenance of buildings and constructions, in particular when surfaces are treated by abrasive (sand) blasting. In 2015, PCB contamination of the river Elbe was caused by the inappropriate removal of paints. Despite enclosure of the working area, approx. 330 kg PCBs was released into the river Elbe. PCB-contaminated sediments moved downstream and reached the Port of Hamburg (distance of 500 km) [75]. In Norway, the sandblasting of PCB paint on a bridge released 1650 kg PCBs to the marine environment and heavily contaminated the sediments [73].

Releases from paints and sealants are also contaminating the terrestrial environment. Releases from buildings [Herrick et al. 2007; 76], pylons [108] and road marking [73] have also resulted in soil contamination [17].

The Swiss experience revealed that, even though the working area during removal of anti-corrosive coatings was securely enclosed, 5–10% of the coating was lost [70, 72].

#### Recycling of PCB-containing oils and wastes and related exposure

PCB-containing oils in transformers and capacitors were not always properly handled and disposed. For Germany, it was estimated that 30–50% of PCBs in closed applications were not appropriately managed [65]. PCB oil can enter oil recycling cycles and has in some cases contaminated feed and related meat and other animal products. The largest and most costly PCB/dioxin food scandal (approx. 1 billion US$) occurred in Belgium in 1999, when PCB oil was accidentally added to a stock of recycled food fat used in the production of animal feeds. Meat and eggs from more than 1500 farms were impacted [18, 19]. Another large meat contamination case resulted from thermal recovery of PCB-contaminated oil for drying of animal feed. Irish pork meat got contaminated and had to be recalled, with an associated damage of 100 million US$ [22, 23].

Still, 14 million tonnes of PCB-contaminated oil and equipment exists worldwide, in particular in developing countries [109]. Such oils are partly recycled in an uncontrolled manner [110], leading to high-exposure risk.

#### Exposure of food-producing animals to PCBs from waste treatment and contaminated soils

At the end of life of PCB-containing products and materials, considerable amounts of PCBs have been released and have contaminated the environment, especially soils [9, 65]. Our survey in Germany discovered contamination of beef or sheep meat from a range of sources related to soil contamination [17]:

- Application of sewage sludge in the 1960s–1970s contaminated with PCBs.^3^ Sites where such sludge was disposed can have elevated PCB (and PCDD/F) levels [111]. At a German reference site, sewage sludge has been applied on soil yearly since the 1950s. In the 1950s, PCB levels were low (< 0.5 ng PCB-TEQ/kg dm). PCB-TEQ strongly increased at the end of the 1960s/early 1970s to around 4.5 ng PCB-TEQ/kg dm [111], when PCBs in open applications were at their peak use. At a German pasture that had been treated with sewage sludge up to the 1960s and 1970s, soil levels were up to 33 ng PCB-TEQ/kg dm, causing elevated dl-PCB levels in sheep liver in a herd grazing on that meadow as well as on uncontaminated pastures [112].
- Contaminated sediment deposits on agricultural land: at a pasture land where sediments were disposed in the past, dl-PCB levels were 3.9–6.4 ng TEQ/kg dm and meat of a beef cattle herd grazing on the land exceeded the EU regulatory limit [52].
- Construction debris from buildings can be contaminated with PCBs and is partly used for landscaping. Such debris was also used on farm land (e.g., for farm tracks), scattered and incorporated into soil of pasture area, contaminating the cattle [17].
- Scrap yards are potentially PCB-contaminated areas [113]. Highest contamination can be expected for sites used to store PCB equipment. However, “normal” scrap yards are also impacted to some extent by PCB-contaminated scrap. In the German study, one beef herd was contaminated by PCBs transferred from the former scrap yard by horse dung stored at the site, leading to PCB contamination of the pasture land and the cattle [17].
- Shredder plants: the release of PCBs from metal shredders remains high. Biomonitoring of rye grass around three shredder plants in Bavaria/Germany showed high levels, up to 130 ng TEQ/kg dm close to the shredder and up to 20 ng TEQ/kg dm outside the plant area. All samples were considerably above EU regulatory limits for feed (1.25 ng TEQ/kg dm) and, therefore, unfit for food-producing animals [114].
- Landfills where partly PCB-containing waste had been disposed: a considerable share of PCBs has been disposed to landfills, in Germany and elsewhere. Areas around landfills can have elevated PCB levels. Chicken eggs from two herds (pooled egg samples) close to the hazardous landfill Eyller Berg had twice the regulatory limit (10.4 and 8.7 pg TEQ/g fat), with major contribution from dl-PCBs. dl-PCBs in soils around the landfill were between 3.1 and 6.6 ng PCB-TEQ/kg dm [115]. This is six–ten times above background dl-PCB levels of German pasture land soil [63], and is sufficiently high to explain the contamination levels in the eggs (see above).
- River floodplains: in the past, PCBs and other POPs have been deliberately released into rivers as a means of “waste management”, leading to sediment contamination [102, 103, 116, 117]. During flooding events, contaminated sediments are translocated to river floodplains [118, 119]. In recent years, floodplains of rivers with historic industrial inputs were identified as a possible PCB and PCDD/F exposure pathway for dairy cows and beef in Germany and the UK [120–122]. dl-PCB levels in soils of floodplains of several rivers in Germany were unfit for cattle grazing or for chicken/egg production [3], and caused contamination of beef and sheep meat [17, 120].
- Open burning: in the Campania region in Italy, large amount of wastes have been dumped and burned in the open. Concentrations of the six indicator PCBs in milk from sheep in the region exceed the European maximum residue limit [123], indicating that the dump sites partly contained PCB oils.

### Sources and entry routes for PCDD/Fs and other unintentional POPs

Article 5 of the Stockholm Convention requires to reduce and, where feasible, eliminate releases of unintentional POPs, including PCDD/Fs, polychlorinated naphthalenes, PCBs, and hexachlorobenzene. Since PCDD/Fs are formed in most processes together with the other unintentionally formed POPs (UPOPs), they are used as indicators for inventories and release reduction efforts for all UPOPs [9]. However, some processes of the organochlorine industry form specific unintentional POPs such as PCBs, PCNs or HCB (see below).

Article 6 of the Stockholm Convention requires the Parties to the Convention to develop an inventory of potentially POP-contaminated areas. Due to this requirement and due to the relevance of historical pollution of soils, sediments and other reservoirs, the UNEP “Toolkit for Identification and Quantification of Releases of Dioxins, Furans and Other Unintentional POPs under Article 5 of the Stockholm Convention on Persistent Organic Pollutants” [9, http://toolkit.pops.int/] contains a chapter for inventory development of PCDD/F and other unintentional POP-contaminated sites and hot spots (http://toolkit.pops.int/Publish/Main/II_10_HotSpots.html).

#### Historic and current release of PCDD/Fs in Germany and impact on soils

In Germany, over the last 200 years, PCDD/Fs in quantities of several 100 kg to tonnes PCDD/F-TEQ have been released into the environment, with the largest releases in the 1940s–1980s from the chlorine and organochlorine industry [6, 8, 124, 125]. For one pesticide factory with a detailed PCDD/F inventory of disposed waste, the PCDD/F-content in waste deposits to landfills was estimated as 333–856 kg TEQ in 53–102 tonnes total sum of PCDD/PCDF [6], which can be compared to the global release of approx. 100 kg TEQ/year from 196 countries today [67]. As early as the nineteenth century, the first chemical factories producing soda (Na_2_CO_3_) from sodium chloride via the Leblanc process, along with bleaching powder from waste HCl, had high PCDD/F releases. For a single Leblanc factory operated from 1840s to 1893, soil assessments indicated a contamination reservoir of 1–10 kg TEQ in the soils [126].

Dated sediment cores from Central European lakes show that the largest amounts of PCDD/Fs were released into the environment in the 1960s and 1970s, and decreased by more than 80% until the 1980s [124, 127–129]. A similar time trend has been observed in Japan, with highest PCDD/F release in the 1960s and 1970s [130] and peak contamination in ocean sediment cores in the 1980s due to a lag of more than a decade for the migration of PCDD/Fs from agricultural soils to ocean sediments [131].

This is confirmed by a German long-term soil study (over a period of 42 years) of PCDD/F and PCB levels in test fields that were continuously treated with sewage sludge or mineral fertilizer [111]. In the 1960s, 1970s and into the early 1980s, the PCDD/F levels in the soil treated with sewage sludge continuously increased and remained at about the same level without further increase since the early 1980s up to 2001 [111]. The PCDD/F levels of the soil treated with sewage sludge were about 8–12 ng TEQ/kg dm and approximately 10–20 times the present background levels in arable soils in Germany [63].

These legacies are now stored in soil, sediments and other reservoirs, such as landfills and contaminated sites [8, 116, 132]. The PCDD/F (and PCB) pollution is ubiquitous in soil and sediments. Soil contamination levels highly depend on historical deposition (e.g., industrial point sources, flood plains, agglomeration areas, sludge entry areas, special contaminated sites). PCDD/F levels in soil range from less than 0.5 ng TEQ/kg dm (lower background pollution level) to 100,000 ng TEQ/kg dm, (e.g., contamination from the Leblanc processes) [126], and up to several million ng TEQ/kg dm where waste containing residues from chloralkali industries that used graphite electrodes in the production process was disposed [133].

After 30 years of release reduction, today’s atmospheric emission of PCDD/Fs in Germany is estimated at about 68 g TEQ/year [10]. This emission level is of minor relevance, since it lies in the sub-percent range of the stored PCDD/F soil load and thus contributes little to the total dioxin inventory and leads to no significant air pollution. The same is true for other industrial countries, which have reduced PCDD/F release from industrial sources over the last decades.

Re-emission/secondary emission rates of PCDD/Fs from soil and other reservoirs into the air seem to be low compared to the current primary emission of 68 g TEQ/year. This can be derived from the annual seasonal cycle of the atmospheric PCDD/F concentration: PCDD/F levels in the air are highest in winter resulting mainly from increased heating. However, desorption from soils and other reservoirs into the atmosphere would be expected to be highest in the warm summer months when atmospheric levels are the lowest [134, 135].

For livestock with outdoor access, soil is the main exposure pathway to environmental PCDD/F. Compared to the PCDD/F reservoirs in soils, which largely reflect the high historic contamination, the current atmospheric PCDD/F release and deposition in Germany and other countries with emission regulations in place is of minor importance for the exposure of livestock and humans via food consumption. In addition, feed and bedding can lead to PCDD/F contamination for all livestock [1, 18, 136]; Fig. 1).

As described above, free-range hens and broilers are sensitive to PCDD/Fs exposure from soil, and low PCDD/F levels in historically impacted soil (from about 3 ng TEQ/kg dm) may lead to exceedance of the EU regulatory limits for chicken/egg. The critical soil contamination is only about three times higher than the PCDD/F background content [63] and is well below the German regulatory limits for soil (100 ng TEQ/kg dm for playgrounds and 1000 ng TEQ/kg dm for residential areas; BBodSchV), and even below the 5 ng TEQ/kg dm in soil suggested by the former Bund/Länder Working Group DIOXINE [137] as safe level.

For dairy cows, a higher threshold for PCDD/Fs in soil may be set. The critical level for milk production can be estimated to be between 17 and 5.3 ng TEQ/kg dm, depending on the cow’s soil intake (3–10%), which in turn depends on the quality of the pasture and the contamination of grass silage by soil [17].

The sources of PCDD/Fs for food-producing animals can be divided into local sources at the farm or pasture and regional sources impacting larger areas (also impacting pasture areas).

The following sections provide an overview of the main PCDD/F emission/exposure sources that have resulted in contamination of food products from animals. The relevance to the exposure of food-producing animals is demonstrated by practical examples. Systematic assessments of these source categories at most sites have not been conducted and contamination is often detected by chance.

PCDD/F contamination sources are structured according to the list in the UNEP “Toolkit for Identification and Quantification of Releases of Dioxins, Furans and Other Unintentional POPs under Article 5 of the Stockholm Convention on Persistent Organic Pollutants”, starting with the most important sources for the historically highest burdens that have often led to PCDD/F-contaminated sites [9].

#### Chlorine-producing industries (UNEP Toolkit category 7)

##### Chloralkali electrolysis

The production of chlorine by chloralkali electrolysis using graphite electrodes, which started in the 1890s, has generated large amounts of PCDD/Fs in waste sludge [9, 133, 138, 139]. Since the 1970s’ graphite electrodes have largely been superseded by metal electrodes, PCDD/F releases have greatly been reduced [9]. The detailed assessment of a chloralkali plant operating since 1890s in Rheinfelden/Germany revealed an inventory of 8.7 kg TEQ in landfills and soils [133]. The historic deposits have been partly remobilized and distributed during city development over nearly a century, resulting in the need for detailed mapping of PCDD/F pollution in the entire city [133]. Studies of eggs in the 1990s showed the highest levels ever reported (up to 514 pg I-TEQ/g fat) [140]. The eggs were 200-fold above the current EU maximum level of 2.5 pg PCDD/F-TEQ/g fat. Eggs produced 2.5 km from a chloralkali and pesticide production site had PCDD/F levels 16 times above EU regulatory limits [30].

##### Leblanc process and related processes

The Leblanc process was used to make soda from 1790 until the beginning of the twentieth century. These facilities produced large amounts of HCl that led to environmental pollution [4, 126]. The HCl was recovered in some factories and chlorine/bleaching was produced via the Deacon or manganese process. In Lampertheim/Germany, high levels of PCDD/Fs, polychlorinated naphthalenes and heavy metals were found on the grounds of a former Leblanc facility [126, 141]. The PCDD/F reservoir in Lampertheim is estimated at 1–10 kg TEQ. The people living on the site had elevated levels of PCDD/Fs in the blood, with the typical PCDD/F profile of the contaminated site [126]. It was not determined whether PCDD/Fs were taken up via livestock or vegetables or another pathway.

There were about 15 Leblanc production sites in Germany and at least 70–100 Leblanc factories were operated in Europe during the nineteenth century, mainly in Great Britain, France and Germany [4]. An investigation of the potential PCDD/F contamination of the former production sites and surrounding areas, including livestock, has not been conducted or documented.

#### Production of organochlorines (UNEP Toolkit category 7)

The highest PCDD/F emissions and reservoirs stem from the (former) organochlorine production. Releases from the production and landfilling of waste from the organochlorine industry were partly in the kg TEQ range, and up to 100 kg TEQ for individual productions [6, 8, 133, 142–145]. A detailed inventory of such waste has only been developed for a few factories. The landfilled chemical waste from a pesticide factory in Hamburg contains 333–854 kg TEQ, mainly from the production of 2,4,5-T and HCH recycling (decomposer process) [6]. Another inventory has been established for the pentachlorophenol (PCP) production in Rheinfelden, with an estimated 7.7 kg TEQ in disposed residues. An inventory of PCDD/Fs has also been developed for a major landfill of wastes from the Basel Chemical Industries, with an estimated content in the 10-kg TEQ range [143, 146]. Soils in the cities of Hamburg and Rheinfelden were contaminated to a considerable extent [133, 147]. In Brazil, organochlorine production has led to the contamination of a larger area, including soils and food [148]. Eggs produced close (2.5 km) to an organochlorine production facility in Russia were 14 times above the EU limit [30]. Contamination of food-producing animals has been documented also for some other organochlorine production sites [20, 21, 30, 133, 140, 149].

PCDD/Fs and other UPOPs are formed in the production of PVC (especially in ethylene dichloride (EDC) synthesis) [9, 21]. PCDD/F contamination, including environmental pollution, has been documented at some production sites [150–152]. Chicken eggs sampled close (2 km) to a petrochemical industrial facility producing vinyl chloride monomer/EDC and PVC in Mexico had PCDD/F levels six times above the EU limit [30]. Lime recovered from an organochlorine industry landfill in Brazil, highly contaminated by PCDD/Fs from EDC catalyst, contaminated citrus pulp pellets sold as animal feeding stuffs to the European Union, thus contaminating cow milk [20, 21].

The production of chlorinated solvents (e.g., tetrachlorethylene, trichlorethylene, carbon tetrachloride) has contaminated numerous sites with UPOPs [153–156]. Solvent production waste contains high levels of several UPOPs, including hexachlorobenzene (HCB), hexachlorobutadiene (HCBD), polychlorinated naphthalenes (PCNs) as “HCB waste” [9, 156–158]. PCDD/Fs are rather a minor contaminant in these wastes [159]. The recent treatment of such “HCB waste” in a cement kiln at too low temperature released part of the HCB and contaminated 332 farms and humans in Austria, resulting in the destruction of approx. 300 cattle, 800 t of milk and 40,000 t of contaminated fodder [160–162]. The unsound management of “HCB” waste from Ukraine at a Polish waste incinerator, including disposal of ash, likely resulted in environmental contamination [163]. An assessment of the impact on the environment and food-producing animals has not been conducted or published.

In industrialized countries, wastes that are highly contaminated with PCDD/Fs and other UPOPs have been burned by some companies since the 1950s, and by most companies since the 1970s. However, in the last 30 years organochlorine industries have moved to developing and transition economies, such as China and India, where companies often face major challenges in managing wastes and environmental contamination [164–167].

#### Use of PCDD/F-contaminated organochlorines (UNEP Toolkit category 7)

The use of pesticides and other organochlorines containing PCDD/Fs (e.g., chloranil, PCP, PCBs, 2,4,5-T, chloronitrophen (CNP) [168–170] has led to PCDD/F contamination of the environment and livestock [8, 18, 171, 172].

The agricultural use of pesticides that contained PCDD/Fs as an impurity [e.g., 2,4-D, 2,4,5-T, PCP, CNP, pentachloronitrobenzene (PCNB)] was one of the most important PCDD/F release sources to the environment. Due to the use of contaminated PCP (pentachlorophenol) and CNP (chloronitrophen) in the 1950s–1980s, approx. 460 kg TEQ has been released into rice fields in Japan, resulting in soil dioxin levels of 30–330 ng TEQ/kg dm [173, 174], considerably above safe levels for raising cattle or producing milk. Some pesticides previously used in agriculture in Germany were also contaminated with PCDD/Fs, however, at lower levels [170] compared to those used in Japan [130, 169]. A survey of PCDD/Fs and PCBs in agricultural soils in Germany shows relatively low levels [63], indicating low-PCDD/F input on agricultural land from pesticide use. However, PCDD/F levels in garden soils in a survey in south Germany (e.g., up to 74 ng TEQ/kg dm) indicated that PCDD/Fs have been released to these soils. In addition to pesticides, other likely sources include PCP use on wood in gardens, ashes from uncontrolled waste incineration in domestic heating systems, and backyard burning. The PCDD/F congener profile in soils and chicken eggs is similar to the PCDD/F congener profile in technical grade PCP [17, 111, 124].

The use of PCP for the treatment of wood, leather or textiles and in agriculture is likely one of the largest dioxin reserves in the technosphere and in the environment. The release of PCDD/F from PCP use in rice fields in Japan was approximately 460 kg TEQ [8, 130]. The Swedish historical dioxin inventory indicates that the amount of dioxin input by impregnation with the wood preservative PCP was 200 kg TEQ in treated woods and 5–50 kg TEQ in soils at the treatment sites [175]. The PCDD/F burden in Queensland/Australia (above all OCDD) arising from the historical use of PCP is also in the tonne range [5]. This shows the order of magnitude of the former applications and the potential current reservoir. The relevance of PCDD/F release from PCP use can also be deduced from the congener profiles in sewage sludge and soils. For example, until the 1990s sewage sludge in Germany, Switzerland or Spain had a PCDD/F congener profile almost identical to that of PCP demonstrating the high impact from PCP [124, 176, 177]. Also, the PCDD/F congener profile in soil and sediment was characterized by PCP [111, 124]. A large amount of OCDD was additionally formed from PCP by condensation in the environment [178, 179].

The impact of PCP-treated wood in stables on dioxin exposure of cattle was demonstrated in a study led by the US Department of Agriculture (USDA) in 2004: out of 158 cattle examined, about 20% had PCDD/F levels in meat above 4 pg TEQ/g fat (8–58 pg TEQ/g fat). All of these cattle where housed in stables with PCP-treated wood, and the PCDD/F profile in the meat correlated with that in PCP [172, 180]. Also, chicken eggs in farms in Germany and Poland were recently contaminated from PCP-treated wood or contaminated soil [17, 181]. In Italy, eggs were contaminated above the regulatory limit due to wood shavings from PCP-treated wood used for animal bedding [171]. The incineration of PCP-treated wood for drying of feed also resulted in contaminated feed and food [18].

In recent years, PCDD/F contamination levels in 2,4-D used in industrial countries seem low (0.12–1.8 ng TEQ/kg) to moderate (160 ng TEQ/kg) [168]. However, there are large differences in the PCDD/F concentration in 2,4-D, depending on the quality of production. Concentrations up to 18,500 ng TEQ/kg have been documented [9]. Therefore, developing countries using low-grade pesticides might still be impacted.

#### Industries using chlorine (UNEP Toolkit categories 2 and 7)

Chlorine-using or formerly chlorine-using industries, such as industries producing paper, magnesium, aluminium or titanium dioxide, have a high PCDD/F emission potential. For example, the historical emission inventory of a single Norwegian magnesium production plant was estimated at 50 to over 100 kg TEQ [182]. For the German magnesium/titanium productions in Bitterfeld, Staßfurt and Aken, high historical emissions have been documented, which have led to contamination of the Mulde, Bode, Saale and Elbe river basins, the Port of Hamburg, and a corridor in the North Sea [116, 183–185]. Alluvial soil on flood plains along 400 km of the Elbe river has been contaminated with high levels of PCDD/Fs. Levels in topsoil were frequently approx. 1000 ng TEQ/kg dm and livestock grazing on flood plains were contaminated with PCDD/F above regulatory limits [122, 186–190]. Soil core layers had levels up to 7000 ng TEQ/kg dm [191].

Titanium dioxide production via titanium chloride using elemental chlorine also has a PCDD/F and PCB emission potential [9, 192], and has led to unintentional PCB pollution in the Delaware River [193, 194].

Paper production with elemental chlorine has historically led to significant PCDD/F pollution [9, 138, 195]. PCDD/F releases were mainly via wastewater and sludge. Sludge residues from paper production have contaminated soils [195, 196]. In one case in Germany, the application of sludge from paper production resulted in PCDD/F levels in the soil up to 150 ng I-TEQ/kg dm, and up to 5165 ng I-TEQ/kg dm in the applied paper sludge material [195].

The historical burden due to polluted sludge disposal and waste from the (former) chlorine industries has not yet been systematically investigated in Germany and other countries [8].

#### Waste incineration (UNEP Toolkit category 1)

Waste incineration plants were another important dioxin emission source. Before appropriate air pollution control and best available techniques (BAT) were required, individual incinerators had an annual PCDD/F release of up to 100 g TEQ/year [197]. These high PCDD/F emissions from waste incinerators in the 1970s to 1990s led to contamination of meat and milk in their surroundings [198, 199]. For soils in the vicinity of a hazardous waste incineration plant in Great Britain, the levels were up to 58 ng TEQ/kg dm [200, 201] and for a US incinerator up to 450 ng TEQ/kg dm [202]. The PCDD/F release of a French incinerator contaminated 365 farms and 6875 livestock, which needed to be destroyed along with 2230 t of milk and 9000 t of hay [203].

After the introduction of emission limits and air pollution control in industrial countries in the 1990s, BAT waste incinerators are no longer significant sources of atmospheric PCDD/F emission [10]. For instance, in Germany, the dioxin release from waste incinerators into air was reduced from 400 g TEQ/year in 1990 to about 2 g TEQ/year today.

In developing and transition countries, standards and monitoring capacity are often lacking and PCDD/F emissions from waste incinerators continue to increase with increasing overall incineration capacity. In a recent monitoring of eggs sampled close to incinerators in an Asian country, PCDD/F levels were 4 and 11 times above EU maximum limit [95, 204].

The total PCDD/F emissions from incinerators are sometimes underestimated by short-term measurements normally conducted during stable operation. However, during the start-up and unstable combustion periods, even state-of-the-art incinerators emit PCDD/Fs in stack gases at concentrations that are up to 1000 times higher than under normal operation [205–207]. Therefore, incinerators and other continuous sources with variation in PCDD/F release into air are better assessed and controlled by long-term sampling [206].

Furthermore, large quantities of dioxin-contaminated ashes (in particular filter ashes) in the order of kg TEQ/year are still generated in Europe. In central Europe, these ashes are disposed mostly in former salt mines in Germany but also on a Norwegian island. (Historical) improper use of such filter ashes has led to PCDD/F contamination of soils unfit for livestock [208, 209]. In developing and transition economies, ash management is a challenge. A recent study has documented environmental pollution and food contamination from incineration ashes in developing countries [210].

#### Metal industries (UNEP Toolkit Category 2)

In addition to incinerators, the metal industry is the most important historic thermal source of PCDD/F emissions [211]. In the IPEN survey, eggs sampled near metal industries in developing countries were frequently several fold above EU regulatory limits [30, 95]. The most contaminated eggs in the IPEN survey, 42 times above the EU regulatory limit, were detected in an industrial area in Egypt with various metal industries [95]. The atmospheric deposition from metal industries and waste incinerators was recently been identified as a main source of PCDD/F in 84 soil samples in Taiwan. With a median content of 10 ng TEQ/kg dm and higher levels in industrial areas [212], soils were unfit for raising free-range chicken/egg.

Sinter plants and copper industry are categorised into Annex II of the Convention as having the highest PCDD/F release risk, while other metal industries are listed in Annex III (Stockholm Convention 2001).

##### Sintering plants

Sintering plants can release high levels of PCDD/F and other UPOPs. Before the introduction of regulatory limits [e.g., 0.4 ng TEQ/Nm^3^, with a target value 0.1 ng TEQ/Nm3 in Germany (according to TA Luft 2002)], the sintering plants had high emissions, up to 100 g TEQ/a for individual facilities [125]. Such high (historic) releases can have resulted in elevated PCDD/F levels in the surrounding soil. The PCDD/F levels in soil within a radius of 5 km from an Italian sinter plant averaged 3.1 ng TEQ/kg dm, with a maximum concentration of 10.3 ng TEQ/kg dm; dl-PCB levels were in the same range [91]. The PCDD/F and PCB contamination from this sinter plant resulted in sheep (meat and liver) exceeding the EU maximum limits and in the destruction of 1600 cattle (see also Sect. 2.4.4) [90, 91, Esposito et al. 2014]. In 2010, grazing was prohibited within a 20 km radius of this sinter plant [92].

##### Copper production

*I*. *Secondary copper production* Copper smelters have a high PCDD/F formation potential because copper is the best catalyst for dioxin formation. High levels in the environment, cattle and milk were found nearby a plant in Austria [213, 214]. The PCDD/F soil content was up to 330 ng TEQ/kg dm.

*II. Primary copper production* Although primary copper production is not classified as a significant PCDD/F source [9], certain processes in primary copper production have resulted in extremely high PCDD/F releases and have generated PCDD/F-contaminated slags. A well-documented case is the 400,000 t of “Kieselrot” ash from a German copper production, which was used on sports fields in Germany in the 1950s and 1960s [215, 216].

##### Electric arc furnace

Electric arc furnace (EAF) can also have high PCDD/F releases, which depend on the air pollution control system and the quality of the scrap used [9]. High releases can be caused by PCB-containing feed stocks (PCB-coated steel parts or PCB-contaminated scrap). For one EAF, PCB emissions resulted in the contamination of fish above EU maximum level in the affected river [87].

##### Aluminum production

*I. Secondary aluminum production* Compared to primary aluminum production, the production of secondary aluminum was a much more important source of PCDD/Fs. For Germany, the annual PCDD/F emission was estimated to be between 25 and 50 g TEQ for the period of 1985–1990 [125].

*II. Primary aluminum production* In primary aluminum production, high loads of unintentionally formed POPs [HCB, PeCBz and octachlorostyrene (OCS)] were generated in the purification process of aluminum with chlorine or other chlorine sources (e.g., hexachloroethane or tetrachloroethane) [217]. Emissions from this process have resulted in high HCB, PeCBz and OCS releases into a river and contamination of fish [217, 218]. The flood plains of this river have not been assessed for potential impact on livestock.

##### Zinc production

Extremely high PCDD/F releases have been detected in the secondary zinc industry during the processing of zinc-containing residues and filter dusts in the roller tube, as well as during hot briquetting [125, 219]. Emissions of up to 194 ng TEQ/Nm^3^ were measured in a plant in Taiwan [220]. Pollution in the soil or livestock around secondary zinc plants have not been published, indicating a lack of assessment of these sites. However, the contaminated zinc oxides from these processes have been used as feed additive, leading to food/meat contamination [24].

Overall, a systematic assessment of PCDD/F contamination in the vicinity of metal industries in respect to food-producing animals has not been done. Heavy metals are likely even more critical emissions from the metal industry compared to UPOPs. In particular, lead pollution has caused serious health impacts, including the death of children in some cases [221–223]. But also cobalt and nickel pollution needs more attention with increasing global use and smelting. PCDD/Fs should become part of a more holistic assessment of pollution and related human exposure and health impacts around metal smelters.

#### Power plants and small combustion plants (UNEP Toolkit category 3)

##### Power plants

Overall, power plants using oil or gas have low PCDD/F emissions, without a significant impact on surrounding soils. However, burning of coal with high chlorine content in coal power plants can result in emissions above 1 ng TEQ/Nm^3^. Furthermore, experience in Poland has shown that the use of copper salts for improved burnout in coal-fired power plants increases PCDD/F emissions 80-fold. In one coal power plant, PCDD/F emissions increased from 0.06 to 4 ng TEQ/Nm^3^, or 2.9 to 972 g TEQ/a [224]. Egg samples near a large coal power plant in Bulgaria had PCDD/F levels 21 times above the EU regulatory limit [95], indicating that some power plants can have high release rates.

Waste wood boilers/incinerators can also lead to high PCDD/F releases due to the use of PCP and copper salts as wood preservatives^4^ [226, 227]. A recent study in Taiwan revealed PCDD/F levels in fly ash from two waste wood incinerators of 99,000 and 38,000 ng TEQ/kg ash [228]. PCDD/F levels were considerably higher than in fly ash from municipal waste incinerators, which are normally below 5000 ng TEQ/kg. Therefore, waste wood must be kept out of biomass boilers that use ash as fertilizer on soils.

The use of waste wood or waste oils for drying animal feed is a source of food contamination. If the feed is directly dried by the flue gas, PCDD/Fs can be deposited onto it. For example, in the Irish pork scandal, PCB-contaminated mineral oil was burnt to dry old bread, which was processed into pig feed [23]. In Brandenburg/Germany, animal feed was dried in the off-gas from the combustion of PCP-contaminated waste wood and was thus contaminated by PCDD/Fs [18].

##### House heating and small combustion plants

After implementing BAT/BEP (best available techniques/best environmental practices) in large thermal PCDD/F sources in industrial countries, the emissions from domestic heating and small combustion plants have become major PCDD/F air emission sources in industrial countries [10, 229, 230]. For instance, this source category accounts for approximately 42% (29 g TEQ/year) of annual dioxin emissions in Germany. Stoves or small wood boilers using exclusively natural wood generally have a low PCDD/F formation/release, which does not result in significant emissions or contamination of ash [226, 231]. However, emissions and ash from small combustion plants in which waste wood or other wastes are incinerated or co-incinerated can have elevated PCDD/F release [226, 231]. Over time, this can lead to soil contamination, and might be a reason for the elevated levels in highly populated areas. For instance, garden soils in densely populated areas in Germany had between 5 and 20 ng TEQ/kg [17, 232] and, therefore, exceeded levels appropriate for raising chicken/eggs.

Furthermore, in recent years, copper salts are commercially marketed in Poland, Germany and other European countries for the “cleaning” of stoves from soot deposits [17, 224]. In a study in Poland, the addition of commercial copper salt to a wood stove has resulted in an increase in PCDD/F emissions by a factor of 1000–10,000, with air emissions of 350 ng TEQ/Nm^3^ and ash above the high Basel Convention “low POPs content” of 15,000 ng TEQ/kg [224]. In Poland, this use is estimated to lead to an increase of PCDD/F air emission from private stoves from 0.04 g TEQ/a to 74 g/a. This can result in locally high dioxin emissions and, if more widely marketed and used, to a significant increase in the PCDD/F emissions of these sources.

The open drying of plants or feed and the smoking of food in the exhaust stream of combustion processes are particularly prone to PCDD/F contamination.

#### Cement plants and other mineral industries (UNEP Toolkit category 4)

Modern cement plants with preheaters are not a significant PCDD/F source [9, 233]. In the past, however, cement plants with high PCDD/F emissions were also measured, with releases of up to 136 ng TEQ/m^3^ and 40 g TEQ in a year [234]. PCDD/F levels in egg slightly above EU regulatory limits were measured in Mozambique and Uruguay [30].

The PCDD/F emissions from the glass or ceramics industry are considered irrelevant and have small PCDD/F emission factors in the UNEP Toolkit [9].

#### Transport and transport sector (UNEP Toolkit category 5)

Dioxin emissions from the transport sector were high at the time of leaded gasoline use. Chlorinated and brominated aliphatic compounds were added to leaded gasoline to volatize the lead. This resulted in the formation and release of chlorinated, brominated and brominated–chlorinated PXDD/F [235]. The dioxin emission factors for unleaded petrol and diesel are small. Since the ban on leaded gasoline, PCDD/F emissions from the transport sector have been insignificant [9]. For instance, the estimated release from the transport sector in Germany was 50 g TEQ/year in the 1980s [125], while only 3 g TEQ was released from transport in 2010, after the end of leaded gasoline [10]. PCDD/F levels in soils along streets were elevated (3.3–52 ng TEQ/kg dm) within the first 2 m, but decreased to 0.9–3.3 ng TEQ/kg at a distance of 10 m [232]. PCDD/F contamination along streets might impact grazing sheep when they move to other grazing areas.

#### Fires and open burning (UNEP Toolkit category 6)

##### Fires

Landfill and dumpsite fires and major building or vehicle fires can release significant amounts of PCDD/Fs [9]. Large fire events can contaminate feed on fields with PCDD/Fs far beyond the EU maximum level of 0.75 ng TEQ/kg 88% dm. After a fire at a metal recycler, levels up to 219 ng PCDD/F-TEQ/kg 88% dm and 37 ng PCB-TEQ/kg 88% dm were detected in vegetation in the neighborhood of the company. At a distance of 800 m, levels of 2.2 ng PCDD/F-TEQ/kg 88% dm were still considerably above the EU maximum limits for feed [236].

In 2011, a fire started at the Chemie-Pack Moerdijk Company in the Netherlands. Large quantities of chemicals were involved in the fire and an enormous cloud of smoke arose and drifted over the surroundings. At a distance of 10 km, the PCDD/F levels in the grass/vegetation were still at 1.1–2.4 ng TEQ/kg 88% dm, and thus significantly above the EU maximum level for feed. This can lead to exceedance of EU maximum levels in food from animal origin (see above and [31, 136]).

A single fire event will usually not lead to significant soil contamination. However, fires at landfill sites and dumpsites have the potential to contaminate the nearby soils and population with PCDD/Fs and PCBs [237]. Landfills and dumpsites frequently burned in the 1960s and 1970s in industrial countries, and are still frequently burning in developing countries, having the highest overall contribution to PCDD/F release in developing country inventories. Eggs sampled around dumpsites exceeded the EU regulatory limit in Kenya (7.6 times), Pakistan (1.5 times) and Senegal (11 times) [30]. The best documented case of large-scale contamination from frequent fires from waste dumps that lead to PCDD/F contamination of the environment, food-producing animals and humans is the Campania region in Italy [238– 240].

##### Open burning (backyard)

Open burning (e.g., in gardens, backyard or along streets) can lead to local PCDD/F contamination, especially when using materials that have a PCDD/F formation potential. These are, for example,

- Incineration of waste wood (PCP treated or treated with copper salts).
- Co-incineration of PVC waste (e.g., agricultural foil/irrigation pipes).
- Co-incineration of pesticide containers.
- Open burning of e-waste or end-of-life vehicle waste.

(Former) open burning is considered a major source for the contamination of free-range eggs above EU regulatory limits detected in the Netherlands [31] and Italy [241].

Sites where e-waste or waste from end-of-life vehicles is burned have high levels of PCDD/F and brominated and brominated–chlorinated PXDD/F [242–245]. Eggs sampled in Thailand at sites where e-waste was recycled involving open burning had PCDD/F levels 33 times above EU regulatory limit and 20 pg TEQ/g fat brominated PBDD/F [33].

##### Forests as a sink for PCDD/Fs and the role of forest fires

Through atmospheric deposition and the ability of leaves and needles to filter PCDD/Fs and PCBs before being deposited to soil, forests function as a sink for PCDD/Fs and PCBs [246]. The background levels of PCDD/Fs and dl-PCBs in German forest soil (16.9 ng PCDD/F-TEQ/kg dm and 8.5 ng PCB-TEQ/kg dm in organic layer) are higher than those of arable land or grassland areas by a factor of 20 for PCDD/Fs and 40–60 for dl-PCBs [63]. These elevated PCDD/F and PCB levels in the organic layer of forest soils are probably the reason for the elevated PCDD/F and PCB levels of game liver and meat (see above).

The PCDD/F emission factors for forest fires and burning of agricultural biomass in the UNEP Toolkit have been reduced by almost a factor of 10 for the 2013 edition, after recent research studies have shown that few PCDD/Fs are formed in forest fires and emissions result rather from the remobilization of adsorbed PCDD/Fs [247]. However, in recent forest fires in California and Australia, hundreds of houses and vehicles burned down leading to high releases of PCDD/Fs considering emission factors for fires of vehicles and buildings [9].

#### End-of-life treatment and landfilling of waste (UNEP Toolkit category 9)

##### Sewage sludge

One factor influencing the PCDD/F and PCB levels of soils is the (historical) input of sewage sludge. PCDD/F levels in sludge-amended soils were 10–100 times higher compared to background soils [112, 248]. In the 1970s to the 1980s, sewage sludge had partly extremely high dioxin loads of several 100 ng TEQ/kg dm up to several 1000 ng TEQ/kg dm. The few sewage sludge data from the 1970s are from Spain, with average values of 620 ng TEQ/kg dm (up to more than 8000 ng TEQ/kg dm) [176]. In the mid-1980s, sewage sludge in Germany still had dioxin concentrations of 200 ng TEQ/kg dm on average [249], which declined to an average of 50–60 ng TEQ/kg dm by the end of the 1980s [250]. Soil that had been amended with sewage sludge up to the 1960s and 1970s had up to 52.7 ng PCDD/F-TEQ/kg dm and caused PCDD/F contamination of the liver of sheep grazing there and on uncontaminated pastures [112].

The PCDD/F contamination in sludge-amended soils depends on the time sewage sludge had been applied (for municipal sludge, 1960s to 1980s) and on specific industries with high (former) dioxin contamination in effluent discharge. Especially the sludge from the pulp and paper industry, the chemical industry (production of chlorine and chlorinated organics) and leather and textile production have had high PCDD/F contamination, leading to potentially significant concentrations in sludge-treated soils [9, 195] that should be assessed.

Today, the dioxin contamination of sewage sludge without specific industry impact is generally below 10 ng TEQ/kg dm in Europe [177, 251]. PCDD/F levels in sewage sludge in Australia were also low, with an average of approx. 6 ng TEQ/kg dm [252]. Municipal sewage sludge in industrial countries seems to largely comply with the German fertilizer regulation for pasture land (8 ng TEQ/kg dm) and other agricultural land (30 ng TEQ/kg dm). In a first screening in Nigeria, levels in municipal sewage sludge were between 9 and 23 ng TEQ/kg dm and 48.2 ng TEQ/kg dm in industrial sludge [253] and, therefore, above the German limit of 8 ng TEQ/kg dm for fertilizer for pastureland and the German regulatory limit for sludge/fertilizer in agricultural applications (30 ng TEQ/kg dm).

##### Disposal of sediments on pastureland

In the past, sediment from river dredging was partly applied to grassland and arable land. Pasture areas contaminated with sediment from the Rhine in the 1990s were identified as contamination source for a cattle herd in 2011/2012, resulting in increased dioxin and dl-PCB levels in the meat of grazing animals (8 pg TEQ/g fat) above EU maximum limit [17, 52].

##### Compost and fertilizer

In the 1980s, compost occasionally had PCDD/F content of more than 100 ng TEQ/kg dm, largely due to PCDD/Fs in pesticides, such as PCP, or additional formation from PCP or other precursor pesticides during the composting process [9, 254, 255]. Therefore, the levels were relatively low compared to sewage sludge and, overall, a moderate contamination potential for soils through historic application of compost can be assumed.^5^ PCDD/F levels were around 10 ng TEQ/kg in early 2000 in Switzerland [256]. In a German monitoring study in 2009, green waste and bio-waste composts from eleven composting plants were found to contain PCDD/F levels averaging 5.3 ng TEQ/kg dm, and showed a decreasing trend compared to 2002 and 2006 [112]. The PCDD/F concentrations in the compost were therefore below the general regulatory limit for fertilizer (30 ng TEQ/kg dm for the sum of PCDD/Fs and dl-PCBs) and in the range of the maximum level stipulated by the Fertilizer Regulation for use on grassland for forage production and on arable land with non-tilling soil cultivation (8 ng TEQ/kg for the sum of PCDD/Fs and dl-PCBs).

A series of commercial garden fertilizers were tested in Germany for contaminants including PCDD/Fs (Öko-Test 2013). Two guano fertilizers contained 5.3 ng TEQ/kg. The PCDD/F profiles were identical to known PCDD/F patterns of clays/kaolinites [257]. Therefore, the dioxins in this sample most likely stem from the clay/mineral part of the fertilizer.

##### Open dumping of wastewater (“Sewage farm”)

Prior to the use of sewage treatment plants, sewage from large cities was partially sprinkled on open areas (sewage farms). In total, more than 30,000 hectares were used as sewage farms in Germany, [258]. Areas used in particular between 1950 and the 1980s may be contaminated with PCDD/Fs and PCBs.

### Footprint of respective PCDD/F and PCB sources and the need for assessment

As mentioned above, a systematic assessment of soil contamination in the vicinity of pollution sources has not been conducted in industrial and developing countries.

A key assessment for individual PCDD/F or PCB emission sources is the area where contamination in soils and grass/feed exceeds the critical levels for food-producing animals, in particular the PCDD/F and dl-PCB levels (< 5 ng TEQ/kg dm) for the most exposure-sensitive livestock (chicken, beef cattle).

The contamination footprint of areas affected by large sources can be substantial. The former PCB production in Slovakia, for example, has been shown to have caused increased human PCB exposure up to a distance of 70 km windward, with food from animal origin considered the most important exposure pathway [81]. Because of high PCB emissions from a large steel plant in Italy, cattle grazing is prohibited within a 20 km radius [90–93]. At two copper smelters treating cables in Germany, high PCDD/F contamination was detected in soil, with levels above 100 ng TEQ/kg dm and up to 10,000 ng TEQ/kg dm within approx. 500 m from the company, and up to 600,000 ng TEQ/kg dm on the company premises [232]. Concentrations decreased to background levels at approx. 2 km distance [232].

The PCDD/Fs released from magnesium/titanium production and chlorine use in the 1940s in Bitterfeld/Germany have contaminated the Elbe River and are transported with sediment into the North Sea. Over 400 km from Bitterfeld towards Hamburg, soil on floodplains has levels of approx. 1000 ng TEQ/kg [116]. In Switzerland, a landfill containing waste from a former capacitor production releases PCBs and contaminates fish in a river over approx. 40 km [86, 87].

In 2015, the removal of PCB paint from a bridge in the Czech Republic released 330 kg of PCBs to the Elbe River. Impacted sediments were moving towards the port of Hamburg, 500 km downstream. This became a matter of concern because sediments contaminated with PCB above a certain limit were no longer allowed to be relocated. As the disposal of these sediments on land was not feasible, access to the Port of Hamburg for seagoing vessels would have been jeopardized [75].

Historic use/disposal of smaller volumes such as scrap yards, substations/transformers or shredder plants can lead to PCB contamination and should be assessed for their pollution footprint and for potential exposure of food animals. Normally, the impact of such sites is mainly localized to possibly a few 100 m radius. However, certain mechanisms can enlarge the polluted area even of such smaller use/disposal sites. A former PCB-contaminated scrap yard in northern Germany was used for storing horse dung before dispersing on pasture. The pasture area was used for beef cows, resulting in dl-PCB meat contamination above EU maximum limits [17].

These examples give a first insight into the footprint of some sources. However, for most PCB or PCDD/F sources, the contamination in the vicinity has not been assessed, and often sources have not even been identified. Relevant sources (e.g., PCDD/F sources listed in Annex II and III of the Stockholm Convention; PCB sources along the life cycle) should be assessed, as well as areas where PCP and other PCDD/F-containing pesticides have been applied in significant amounts.

### Management measures for selected livestock

For farms where point sources of PCDD/Fs or PCBs caused contamination of food, the management measure consists of removing the source or excluding or restricting access for livestock. This is what was done in the 1980s following the discovery that PCB paints in feed silos caused the PCB-contamination of milk exceeding the former German regulatory limit for ndl-PCBs [3, 99]. At that time, a great number of farms were screened and the PCB-painted silos were removed [29]. In the case of cattle contamination on a Swiss farm by PCB-containing wall paints [107], the paints were professionally removed and the levels in meat decreased [259].

For herds grazing on areas where the soil or vegetation is the cause for the exceeding of the limit in meat or milk, soil removal is normally not an option due to the high cost. In highly contaminated areas, the production of food might need to be stopped. However, in the German survey, 90% of veal calves from beef cows not complying with the EU maximum level exceeded the regulatory limit for the sum of PCDD/Fs and dl-PCBs only by less than 20% [17]. Therefore, management measures to reduce exposure in impacted areas might be sufficient for further livestock farming. Management measures have been developed [260] for the highly contaminated floodplains of the Elbe River [122, 186]. Studies have shown that it is possible to decrease PCDD/F levels in beef by feeding non-contaminated feed in the fattening phase before slaughtering [261]. A similar approach was found for reducing PCBs in pigs [59] and PCDD/Fs and PCBs in sheep [53]. For a suckler cow herd it has been found that TEQ values in the meat decrease after weaning (Fig. 4; [52]). Extending the duration between weaning and slaughtering might, therefore, be a relevant factor for reducing the PCB and TEQ levels in meat from beef cattle. Another option is the reduction of the suckling time, which has already been applied on the Elbe flood plains [261]. These reductions are mainly due to the increase in body mass diluting the load from the higher PCB intake when suckling milk (Fig. 4) or from the high exposure to PCDD/Fs on contaminated land before fattening in the stable [261].

The selection of appropriate feed and cultivation methods on impacted fields can reduce exposure for livestock. For example, it is possible to grow maize (whole plant; high cut technique) on contaminated soils with low transfer of pollutants to the feed [45]. Optimizing the harvesting technique (e.g., cutting heights) of fodder on contaminated soils can also reduce the dioxin contamination of grass/green fodder from soil [45]. Free-range areas should have a continuous soil vegetation cover to reduce exposure to PCDD/Fs via soil while foraging [10]. Additional concentrate feed can also reduce the grass/soil intake and therefore the overall exposure.

With these moderate management measures, for the vast majority (90%) of the slightly impacted offspring from suckler cow herds it seems feasible to bring TEQ levels below the analytically guaranteed EU maximum limits.

These are encouraging examples for further assessing what management measures might be applicable for other contaminated areas and what type of livestock can be addressed by which management measures. Further research is needed on the suggested critical dl-PCB (and PCDD/F) levels in soil and feed with regard to the resulting levels in meat and other animal products. Some initial assessments have been made for sheep and goats [53, 58]. However, there are no studies on pigs housed outdoors or fed with feedstuff with a high soil content.

Due to the increasing demand for animal products from sustainable species-appropriate livestock farming, there is an increasing trend of housing farm animals outdoors. Unless measures are taken to reduce exposure (e.g., by monitoring soils), there will likely be a number of instances of animal-origin food exceeding EU maximum contaminant levels.

More experimental data are needed for solid recommendations.

For farms producing chicken/eggs with levels above regulatory limits, a guidance for assessment and management has been developed in Germany. Based on the BMU Guide for Poultry, Cattle, Sheep and Pig Keepers (chapter 8 of BMU [10]), a project group led by the Lower Saxony Ministry of Food, Agriculture, Consumer Protection and Rural Development developed a leaflet with recommendations for affected chicken/egg farms [262]. This material is used by the competent food control authorities and by farmers for the identification of possible sources and, when regulatory limits are exceeded, for exposure source identification and mitigation.

If applicable, the following measures should be taken into account [48, 262]:

- Visual inspection of stables and free-range areas (assessment of possible exposure to PCDD/F and PCB sources on the basis of a questionnaire for farm analysis for chicken keepers).
- Removal of possible point sources and hot spots on the site.
- Expert examination of the soil and, if necessary, proper replacement of contaminated soil.
- Whole-quality feeding (full supply of feed including minerals).
- Feeding in stables or on paved areas.
- Covered outside area.
- Restricted access to free-range areas.
- Closed vegetation cover on free-range areas.

Since farmers do not normally have the knowledge and experience for assessing PCDD/F or PCB sources and reducing exposure, support from authorities, institutions or consultants is needed. In some parts of Germany, federal state-level task teams have been established to support affected farmers. The following possible supporting measures should be evaluated by the authorities and offered, as appropriate, to farmers:

- Provision of practical examples of potential sources of contamination and general advice for preventive measures for farmers—and in particular for holders of backyard poultry flocks.
- Provision of elaborated instructions for affected farmers (for example, leaflet of the Chamber of Agriculture of Lower Saxony).
- Advice for farmers with food products (eggs, meat, milk) above the EU maximum level.
- Support in the review of farming practice.
- Support for the expensive PCDD/F and PCB monitoring of affected farms (animals and environment/sources).

The cost for the management measures should be covered by the polluter considering the polluter pays principle and the extended producer responsibility (see below).

### Some further relevant considerations to appropriately address the exposure of food-producing animals

#### Impact of climate change on POP-related contamination

In the previous chapters it has been shown that grass/feed growing on flood plains can lead to POP exposure for livestock. But also, the quality of the pasture is a relevant factor for the contamination of livestock and livestock products. Both factors are related to weather conditions, and change in precipitation and increased flooding can be impacted by climate change:

##### Change in precipitation and increase in ingestion of soil by cattle during grazing

As described above, cattle have a certain intake of soil when grazing. The amount of soil ingested by cattle during grazing is closely related to the condition of the soil and the grass cover. For areas with good grass cover, a minimum share of 3% soil in ingested pasture grass (88% dm) is expected (the share of soil intake refers to the weight of ingested grass recalculated to 88% dry mass) [10, 50]. Soil uptake during grazing can increase at low vegetation coverage. 10–20% of total ingested dry matter may consist of soil. Dry weather and droughts, therefore, have a significant impact on soil intake and lead to higher exposure to soil contaminants. Wet weather can also lead to an increase in soil intake. When grazing areas become wet and muddy a higher share of soil/mud particles is transferred to vegetation due to splashing water and trampling damage caused by grazing livestock. Higher soil intake already results in exceedance of regulatory limits at lower levels in soil. Therefore, with climate change, exposure to soil will increase due to droughts and high precipitation, and livestock will become more exposed to soil pollution in future. Extensive livestock farming with elevated soil uptake in climate-impacted areas will likely need even more stringent soil limits.

##### Increased pollutant release during flooding events and increased transfer to floodplains

In rivers contaminated by former industrial emissions, more frequent and extensive flooding [263] increases the distribution of contaminated sediments to floodplain soils leading to increased exposure risks for grazing livestock [264]. Increased flooding can also result in the release of POPs and other pollutants from reservoirs like landfills [265, 266] and increase exposure of food-producing animals in the vicinity.

Furthermore, increased sea level rise and erosion of coastal areas result in pollutant releases from landfills located next to the shoreline. Damage resulting in pollution was recently revealed for more than 1000 landfills in the UK [267, 268] and likely increased contamination of fish and seafood.

#### POP input and degradation in soils over time

The half-lives of PCDD/Fs and PCBs in soils are decades to more than a century in the Central European climate [126, 269]. Therefore, PCDD/F and PCB contamination in soils will be relevant for decades to come and must be appropriately addressed and managed. For tropical soils, PCDD/F degradation, in particular of lower chlorinated congeners, might be faster.

More robust data on the soil half-lives of PCDD/Fs and PCBs are needed for predicting future development and for modeling future levels in soils considering ongoing deposition of PCDD/Fs and PCBs.

#### Compilation of fingerprints of PCDD/F and PCB sources

An important aspect in the identification of PCDD/F and PCB sources is the assignment of source profiles (“fingerprint”, congener pattern) [270, 271]. The PCDD/F or PCB fingerprint in environmental media or feed and food can help identify contamination sources and pathways [21, 25]. Congener profiles differ in emissions from certain industries, in specific chemicals or other sources that cause, for example, the contamination of feed. The fingerprint also allows environmental contaminants in soils, sediments, or feed and food to be assigned to specific processes or chemicals. This requires a comprehensive database of source profiles. The German EPA had developed a database, initially launched for PCDD/Fs, with a wide range of environmental matrices (soil, air, sediments), food and a few processes from the technosphere. Within the scope of the R&D project of the German Environment Agency, current available source patterns in the database were assessed, and relevant PCDD/F and PCB source patterns from the technosphere were compiled and entered to the POP–dioxin database. These include for instance industrial thermal sources, other industrial processes with high formation potential, chemicals and mixtures such as pesticides and color pigments [17].

#### Contemporary developments

In recent years, biogas production has strongly increased. The residues from biogas production are often distributed on agricultural land. To some extent, industrial sludge is added/used in biogas production such as sludge from pulp and paper [272, 273]. Depending on the production process, pulp and paper sludge can be contaminated with PCDD/F (if elemental chlorine is used) or with per- and polyfluorinated alkylated substances (from specific surface-treated papers) [195, 274, 275]. This can lead to large contaminated areas, as recently demonstrated for a PFAS-impacted pulp and paper mill sludge added in a composting plant [274, 276].

#### Possible future change of TEF values for PCBs and reduction of the TWI for the TEQ

The TEQ from dl-PCBs is driven by one congener (PCB-126) representing normally > 80% of the PCB-TEQ in food. Data from human-cell systems from the EU-SYSTEQ project indicate the possible re-evaluation of the toxic equivalency factor (TEF) for PCB-126 (0.1). The TEF expresses the toxicity of individual PCDD/F and PCB congeners in terms of the most toxic dioxin compound, 2,3,7,8-TCDD, and is derived from mouse and rat experimental data for dioxin intake. In studies on selected human cells, PCB-126 had a systemic TEQ activity lower by a factor of 10 or more compared to the current TEF of 0.1. Since the TEQ contribution of PCB-126 in beef from suckled cows is usually greater than 80% of the TEQ, a PCB-126 TEF by a factor of 10 would have a decisive influence on the overall PCDD/F-PCB-TEQ (reduction by approx. 50%). For example, for beef meat sampled in Germany, most non-compliant samples had levels only slightly (< 25%) higher than the EU maximum limit. A reduction of the TEF for PCB-126 by a factor of 10 would result in a significant reduction in the number of meat samples above the EU maximum limit. Thus, further assessment of PCB-126 is needed.

On behalf of the European Commission, EFSA established a Working Group to assess the risks to human and animal health related to the presence of PCDD/Fs and dl-PCBs in food and feed [42]. All available toxicological, epidemiological and toxicokinetic studies in the open literature were evaluated. Adverse effects of PCDD/Fs and dl-PCBs in humans were assessed and the TWI was revised [42]. The EFSA scientific opinion will be published during 2018 and the TWI for PCDD/Fs and dl-PCBs will likely be reduced.

In PCB risk assessments, it should also be considered that in 2013 the International Agency for Research on Cancer (IARC) has categorized PCBs in the highest cancer class (Group 1, carcinogenic to humans) [277, 278], with a need for minimizing exposure. PCBs are immunotoxic and neurotoxic at very low levels [41], which should be taken into account.

## Conclusions and recommendations

According to FAO and the Intergovernmental Technical Panel on Soils (ITPS), soil pollution is one of the ten major soil threats identified in the 2015 Status of the World’s Soil Resources report [35] and subsequently addressed in the FAO Voluntary Guidelines for Sustainable Soil Management (VGSSM) [279]. PCDD/Fs and PCBs are only two of the pollutant groups—however, two of the best assessed with respect to release sources and contamination.

Within the framework of the R&D project, the research and regulatory action needs regarding the problem of livestock products contaminated with PCDD/Fs and PCBs and the reduction of emission and exposure were compiled [280]. Some priority action needs are mentioned here. More details can be found in Weber et al. [280].

### Need for regulatory activities

#### Upper levels for contaminated soil and feed

In general, soil pollutant levels are assessed in Germany using the precautionary, assessment and action/restriction values of the Federal Soil Protection Ordinance (BBodSchV) [50]. For PCDD/Fs, the BBodSchV gives only action/restriction limits for the soil–human pathway. To prevent children’s exposure, the BBodSchV gives an action/restriction limit of 100 ng I-TEQ/kg dm for children’s playgrounds and of 1000 ng I-TEQ/kg dm for all residential areas. The critical PCDD/F levels for laying hens are thus approx. 300 times lower than the values for residential areas, where chickens are kept mostly in backyard with associated consumption of eggs. Currently, the BBodSchV is being revised and will be extended to the so-called “Mantelverordnung”. In the ministerial draft of the “Mantelverordnung” from February 2017 the action values for children’s playgrounds and residential areas now refer to the sum of PCDD/Fs and dl-PCBs (PCDD/F–PCB-TEQ (2005)) but numerical values will remain unchanged [281].

In the ministerial draft for the amendment to the BBodSchV, an assessment value of 15 ng PCDD/F-TEQ (2005)/kg dm for grassland was set for the soil–plant pathway [281]. For laying hens, this value would be about three–five times too high.

The former German Bund/Länder Working Group DIOXINE published guidance values for soil. For soil with levels below 5 ng I-TEQ/kg dm, it suggested no restrictions for agricultural and garden use [282]. Use of soil with 5–40 ng I-TEQ/kg dm for food and field crops is unrestricted, but a restriction of grazing or abandonment of free range is recommended for subsistence farming. Above 40 ng I-TEQ/kg dm, soil-based livestock farming and cultivation of near-surface growing crops, fruit and vegetables are to be avoided [282]:

The maximum value for PCDD/Fs of 40 ng I-TEQ/kg dm for livestock farming recommended by the former working group AG DIOXINE is also too high for beef production via beef cow herds. Critical levels in the meat are expected at PCDD/F soil levels of about 7–20 ng TEQ/kg dm [17]. For dairy cows, a critical soil level has been established by Hoogenboom [136]: Considering 3–10% soil intake, the EU maximum level for PCDD/Fs in milk might be reached at PCDD/F levels of 17–5.25 ng PCDD/F-TEQ/kg soil, respectively. Since it is important that soil levels below the existing guidance values for soil should not lead to exceedance of maximum limits for the production of food from animal origin, there is a need for updated regulations.

The current action value for dl-PCBs in feed of plant origin (e.g., grass, hay, silage) is 0.35 ng PCB-TEQ/kg 88% dm. This is too high for calf from beef cow herds already reaching dl-PCB limits at 0.2 ng TEQ/kg dm or below, given additional PCB input from soil (Fig. 2). The PCDD/F–PCB-TEQ maximum level for feed (1.25 ng TEQ/kg 88% dm) is approx. six times higher than dl-PCB levels critical for suckler cow herds.

Parts of the European population are exceeding the TWI for the sum of PCDD/Fs and dl-PCBs as well as the tolerable daily intake (TDI) for PCBs via consumption of food (see Sect. 2.1). Guidance values for soil should not only guarantee compliance with EU maximum limits for food but should also provide protection in accordance with the TDI/TWI. The pathway “soil–(feedstuffs)–livestock–animal product–human” has not been considered yet when deriving limit values for soil. However, the R&D project has shown this route of human exposure to be the most critical exposure pathway for PCDD/Fs and dl-PCBs from soil to humans [17]. These low soil levels for livestock farming have to be included in the Soil Protection Ordinance.

#### Regulatory framework for the management of PCBs and hazardous substitutes in open applications

While PCBs in closed applications, in particular transformers and capacitors, are addressed by regulations in industrial countries, and increasingly in developing countries with the implementation of the Stockholm Convention, PCBs in open application are often neglected. However, open applications are relevant to exposure, and levels in the population^6^ are particularly elevated in countries that produced (and extensively used) PCBs, including in open applications, as demonstrated by the WHO human milk studies (e.g., Czech Republic and Slovakia, Germany, Italy, Russia, United States). These PCB-producing countries and countries having extensively imported PCBs and used in open applications should develop an appropriate regulatory framework including requirements of inventorying PCBs in buildings. Sweden has developed an inventory of PCBs in buildings and could serve as example for a suitable inventory and management of PCBs in construction.

Furthermore, hazardous alternatives to PCBs used in open applications, such as PCNs and SCCPs, need also an appropriate regulatory framework after they were listed as POPs in the Stockholm Convention in 2015 and 2017, respectively.

#### Controlling and limiting further contamination input to soil

Effective regulatory frameworks are needed to protect soils from further pollution input of POPs and other pollutants. Such a framework needs to regulate air emissions from industries and incinerators and should require the use of best available techniques and best environmental practice (BAT/BEP) for such facilities as stipulated by Article 5 of the Stockholm Convention. In addition, the amendment of soils with contaminated sludge, ashes or sediment needs to be avoided and restricted. To achieve such a protection, regulatory frames such as a regulation for fertilizer and other materials used for soil amendment is needed. The German fertilizer regulation includes limits for some POPs including PCDD/Fs, PCBs and PFOS. The limit for applying fertilizer to pasture land is 8 ng TEQ/kg dm for the sum of PCDD/Fs and PCBs [283].

#### Information to farmers and impacted population

Information on soil and feed contamination available to authorities should be given to the local farmers who produce fodder/feed materials or keep animals outdoors. This should include also floodplains from industrially impacted rivers.

Suspected or potentially impacted areas should also be assessed by the authorities or the respective polluter to support farmers using potentially impacted land for feed production or for extensive animal farming. The farmer should be informed about the levels of contamination and receive support towards an appropriate management of food production in these areas, or towards switching to other uses in case (certain) food production is not feasible.

Similarly, people living or working in PCB-contaminated buildings (e.g., schools, universities, and kindergartens) should be informed about the existence and magnitude of the pollution (“Right to know”). Appropriate guidance regarding the duration of use of the buildings should be given along with this information.

#### Damage and cost compensation by the responsible polluters

While farmers are normally not the polluters, they are currently considered responsible for safe feed and food production and often bear the costs when maximum contaminant limits are exceeded and the food product is restricted on the market and needs to be destroyed. However, the cost of the damage (impacted animals, polluted area, cost of management measures) should be covered by the polluters considering the polluter pays principle and the extended producer responsibility. Farmers are not responsible for the past use of PCBs. Authorization and control of hazardous chemicals are regulated by law, and these chemicals were produced and released by companies and continue to be released via their products.

Furthermore, farmers are normally not responsible for the PCDD/F contamination (e.g., of areas impacted by industrial emissions) except for, e.g., open burning in the backyard or non-authorized use of waste sludge on pasture land.

In all cases where farmers were not responsible for the environmental pollution and feed or food contamination, they need to be supported by the government and financially compensated by the polluters and producers or the government.

### Research needs

A range of research needs for improving food safety have been identified within the R&D project [280]. These include, for example,

Assessment of PCB- and PCDD/F-contaminated sites and potentially contaminated areas, especially of soils where animals are housed outdoors or feeds are produced:

- Investigation of areas contaminated with PCBs and the impact of remediation of buildings and constructions.
- Footprint/extent of PCDD/F- and PCB-contaminated sites from industrial sources.
- Relevance of PBDD/F and PXDD/F for selected contaminated sites.
- Long-term perspective of PCB and PCDD/F soil contamination and degradation.
- Monitoring of soils and feed of potentially contaminated areas.
- Monitoring of exposed food-producing animals on (moderately) contaminated areas.
- Measurement and modeling of the spatial distribution of PCBs and PCDD/Fs in soil contamination around (historical) point sources.

Research needs on PCBs in open applications:

- Inventory of remaining amounts of PCBs in open applications.
- Open PCB applications and their relevance as point sources on farms (paints in silos, feed stores, silage bunks, manure pits).
- Assessment of PCB contamination of recycling cycles.
- Long-term emission of PCBs from sealants, paints and coatings.
- Risk of increased PCB indoor exposure due to increased insulation of houses.

Research needs for PCDD/F and PCB release from emission sources:

- PCDD/F emission from copper salts for the purification of small furnaces.
- PCDD/F releases of ashes from biomass combustion and related levels and risk.
- Modeling PCB and PCDD/F emission around point sources.

Analysis and validation of exposure estimates for free-range poultry, including eggs:

- Further assessment/verification of the extent of exposure for free-range chicken/broilers with regard to transfer of contaminants into meat and eggs.
- Examination of critical PCDD/F and PCB levels in soil and factors influencing uptake by chicken.
- Examination of free-range broilers, ducks and other poultry.

Assessment of the extent of exposure and problematic soil levels of food-producing animals to soil contamination, where data are weak (pigs, horses, game, ducks/geese).

Investigation of the fate of pollutants within the animals:

- Metabolism.
- Best indicator: Which organ reflects body load best? Selective accumulation in liver?
- Development of methods to measure the level of contamination in animals without slaughtering (e.g., in fatty secretion).

Options for management measures for reducing PCDD/F and PCB levels in affected cattle/sheep, and their effectiveness:

- Influence of the suckling period and the period between weaning and slaughter (withdrawal time).
- Effect of supplement feed with low PCB and PCDD/F levels.
- Breeds with low PCB/dioxin accumulation potential.
- Implementation of adapted pasture management strategies.

Options for management measures for reducing PCDD/F and PCB levels in laying hens, and their effectiveness:

- Compilation and validation of management measures.
- Review and further development of management measures on impacted flocks.

Research needs for PCDD/F, PCB and POP/PBT reservoirs and the impact of flood events:

- Assessment of contamination of river sediment, floodplains, and other flooded areas.
- Inventory of PCBs, PCDD/Fs and other POPs/PBTs in landfills, and assessment of flooding and mobilization risk.
- Potential impact of flooding events on livestock (mobilization and remobilization of PCBs, PCDD/Fs and other POP/PBT substances in sediments).

Systematic assessment of other POPs regarding their life cycle similar to PCBs (Fig. 5) and their risk for environmental and food contamination:

- The use of PCB alternatives, in particular SCCPs (listed as POPs since 2017), MCCPs [284], and the related exposure of humans and food-producing animals.
- PCB alternatives (in particular SCCPs and PCNs) and their impact on recycling cycles and potential exposure of livestock and humans within the circular economy framework.
- Life cycle of brominated flame retardants, their releases and resulting exposures.
- Life cycle of per- and polyfluoroalkyl substances (PFAS), their releases and resulting exposures.

### Inventory of PCDD/F and PCB-contaminated areas and environmental matrices

#### Inventory of PCDD/F- and PCB-contaminated areas

Due to historical pollution, PCDD/F and PCB levels in soil, sediments and other reservoirs should be inventoried and assessed for exposure potential. Soil PCB and PCDD/F levels critical for potentially exposed livestock should be used as a benchmark for assessing areas potentially contaminated with PCDD/Fs and PCBs. For food safety and for minimizing exposure, contaminated sites should by systematically screened and appropriately secured, managed and possibly remediated. Such an assessment could also clarify what areas are safe for producing feedstuffs and food products from animal origin (and plants/vegetables).

An assessment of POP-contaminated sites in the 182 countries that have ratified the Stockholm Convention on Persistent Organic Pollutants (the Stockholm Convention) is required as part of the implementation process. Article 6 of the Stockholm Convention requests that parties “Endeavour to develop appropriate strategies for identifying sites contaminated by POPs”. The development of an inventory of potentially PCDD/F-contaminated areas is, therefore, necessary, both for meeting the obligation to implement the Stockholm Convention and for food safety.

To support countries/parties in their efforts, inventory guidance documents have been developed within the Stockholm Convention or by UN organizations. To facilitate such assessments, the UNEP inventory guidance document for PCDD/Fs and other unintentional POPs contain a chapter on contaminated sites inventory in chapter 10 of the UPOPs toolkit (http://toolkit.pops.int/Publish/Main/II_10_HotSpots.html). Another guidance document has been developed by United Nations Industrial Development Organization [285].

#### Inventory of PCBs in open applications

One-fifth of the global PCB use was in open applications [109, 286, 287]. In West Germany, open use accounted for about one-third or more of the total PCB use [65] and was possibly the highest in the world. It is estimated that the majority (> 50%) of PCB-containing sealants and paints/coatings are still in use today [76, 288]. Open PCB applications can be found in the everyday environment in buildings and constructions. A systematic inventory of PCBs in buildings has only been developed/required in Sweden. In Germany, there is no national inventory of PCBs in buildings, not even for state-owned properties.

In industrial countries with large historic use of PCBs in open applications, the largest releases of PCBs stem from this source. The need for developing a regulatory framework for the inventory of PCBs in buildings and structures has been recognized [280, 289]. Inventory and control of remaining PCBs in open applications have a high priority for industrial countries, to lower and control current PCB impacts on the atmosphere and grassland, and the resulting burden on farm animals. If and where an inventory of specific open applications is important and useful needs to be determined. For example, it is estimated that PCB paints were used in 20% of outdoor swimming pools in Switzerland [74]. Other applications with potential relevance are for instance paints/coating used for road markings, electricity pylons, silos, stables, and liquid manure pits [17, 73].

Since chlorinated paraffins were a major substitute for PCBs in these open applications, and since SCCPs are listed as POPs in the Stockholm Convention as of 05/2017, they should be included in such an assessment of open applications and related management.

#### Inventory of PCDD/F- and PCB-impacted sediments

PCDD/F- and PCB-contaminated sediments are considered important reservoirs [116, 144]. However, only a few detailed inventories and mass flows have been compiled so far, to give an insight into the current total PCDD/F and PCB contamination of some water bodies [144, 290], and an estimate of the future impact of sediment loading in river deltas or floodplains. A detailed inventory and substance flow analysis might help to predict trends in fish contamination or estimate the burden of food-producing animals grazing on floodplains.

### Reduction of PCB and PCDD/F release, management and reduction of exposure

The contamination of food from animal origin and the low levels of PCDD/F and PCB in soil at which food-producing animals become contaminated above EU maximum limits highlight the need to further control and reduce releases into the environment.

#### PCB in open applications and reduction of emissions

For industrial countries with significant former use of PCBs in open application, the remaining PCBs, mainly in the construction sector, have a significant emission potential as demonstrated for Germany (7–12 t PCB/year). Furthermore, extreme high release occurs if paints or sealants are sandblasted or otherwise removed without proper technology or waste management measures [73, 75, 76]. Even with BAT control, 5–10% of PCBs are released into the environment when removing paints from outdoor construction [72, 70].

Therefore, inventorying and control of remaining PCBs in open applications is important for lowering and controlling current PCB impacts on the atmosphere and grass, and the resulting burden on food-producing animals. PCBs from open applications can also impact construction debris. The recycling/reuse of such materials around farms or grazing areas can result in exposure. Other POPs also need to be inventoried, controlled and managed, and their exempted uses phased out as soon as possible (see below).

#### Management of remaining PCBs in closed applications

While PCBs in transformers and capacitors have already largely been disposed in the EU and a range of other industrial countries, developing countries are still struggling with inventory, management and exports for destruction. The most recent global inventory estimated that 14 million tonnes of PCB-contaminated equipment and waste oils must still be managed and destroyed [109]. Contamination from PCB use and inappropriate end-of-life management and the associated long-term impacts from contaminated soils, sediments and feed highlight the urgent need to progress faster compared to the past 10 years of Stockholm Convention implementation, in particular when considering that PCB oils are currently recycled in part for cream and pomade, underbody protection of cars or welding operations [110, 291], and PCB-contaminated equipment is sold for recovering of metals in many developing countries.

With treatment costs of 1000–5000 USD per tonne (including packing, transport, and destruction), management of the remaining PCB equipment and contaminated oil would amount to an estimated global cost of 14–70 billion USD. The overall GEF funding available for POP management is less than 1 billion USD for GEF 7. Therefore, additional funding for PCB management is needed. Following the extended producer responsibility principle [292], the original PCB producers should help manage and destroy their former products.

#### Reduction and minimization of PCDD/F emissions

**Fig. 6:**
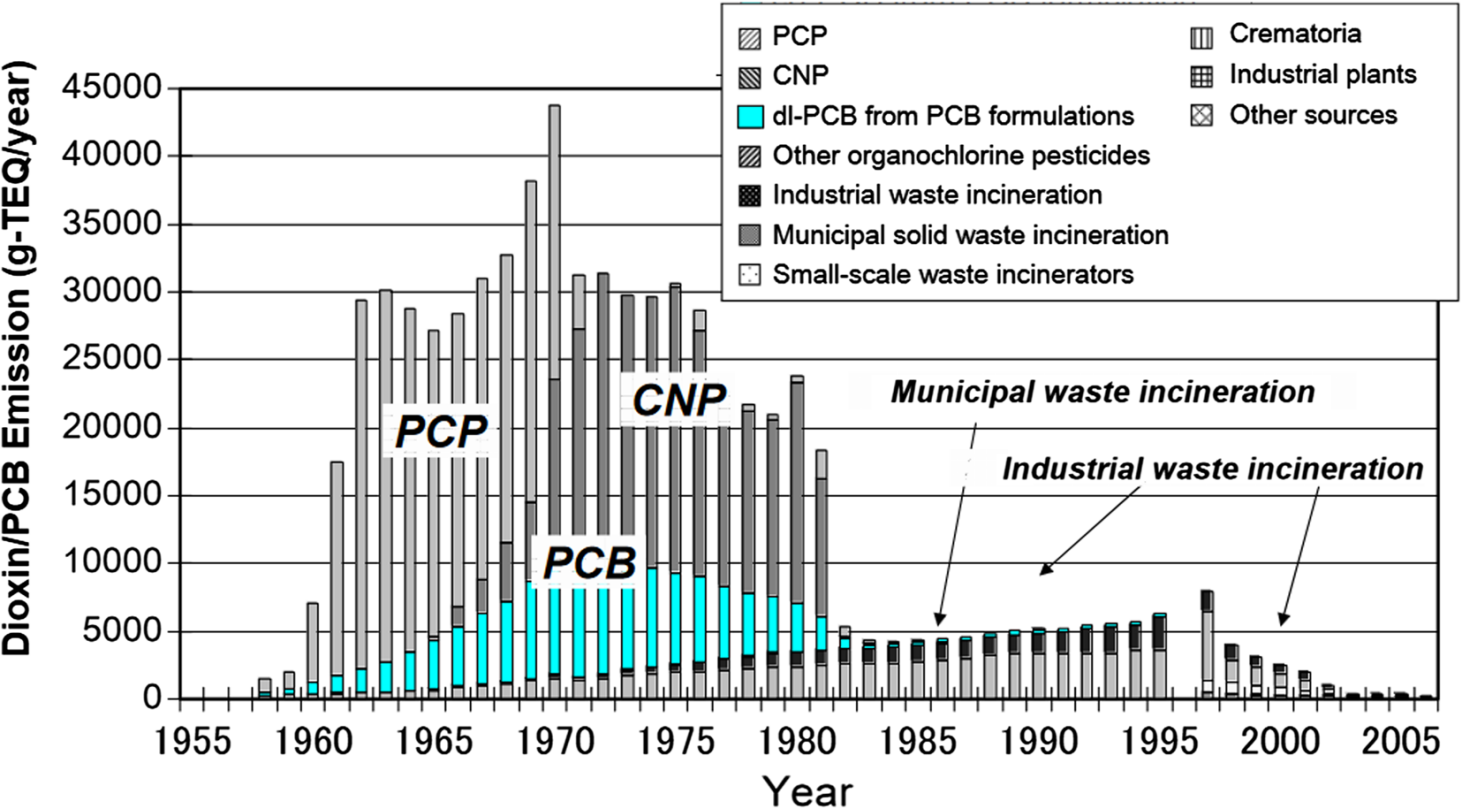
Historic release of PCDD/Fs into the environment of Japan [295, 130]

As mentioned above, the PCDD/F emissions to air have significantly decreased in Germany, Japan and most industrial countries in the last 30 years (Fig. 6; [10]), and are mostly below levels of concern. However, one increasing source of PCDD/F release in European countries is the use of copper salts for burning off deposited soot in wood and coal stoves. This is basically facilitating and optimizing PCDD/F formation and release by degradation of soot and PAHs by de novo synthesis using the best catalyst (copper) for PCDD/F formation [293, 294]. The salt is broadly marketed and increases PCDD/F release from wood stoves 1000–10,000 times [224].

The situation in developing countries is different. While PCDD/Fs are considered within the framework of the Stockholm Convention and PCDD/F inventories have been developed in most developing countries, the release of PCDD/Fs has not been reduced due to the lack of improved waste management measures as well as the lack of capacity and funding to implement best available techniques and best environmental practice (BAT/BEP) in industries. A range of updated PCDD/F inventories in developing countries even show an increase in PCDD/F release due to increased consumption and release in uncontrolled incineration processes such as open waste burning [291, 296]. In addition to landfill fires, waste is often burned in backyards and along streets where chicken, goats and cattle forage for food, leading to exposures. Large areas are at risk of becoming contaminated over time leading to exposure, similar to the Campania region in Italy [239].

#### Reduction of PCDD/F and PCB intake by reducing food of animal origin and co-benefits

Meat and dairy products (milk) and fish are major sources of PCDD/F and PCB intake with lower impact from vegetables [12]. Therefore, an overall reduction of food from animal origin reduces the exposure to these POPs. PCDD/F, PCBs and other accumulated carcinogens in meat are considered a cause of increased cancer rates with increased red meat consumption [297, 298]. A reduction of red meat consumption (in particular processed red meat), therefore, also reduces the associated cancer risk but also other meat-related diseases such as cardiovascular disease, type 2 diabetes, as well as total mortality, in both men and women [298–300]. A more vegetable- and fruit-based diet reduces the overall mortality [301].

Furthermore, the reduction of meat consumption also significantly reduces the ecological footprint of food [302]. Therefore, overall the reduction of high meat consumption can combine reduced health risks and reduced ecological footprint for a more healthy and sustainable nutrition [303, 304].

### Assessment of emerging POPs of concern

Within the R&D project for the German Environment Agency, the focus was on PCDD/Fs and PCBs since they are regulated in foodstuff in the EU along with a few POP pesticides [13, 14]. Most other POPs, in particular newly listed industrial fluorinated and brominated POPs, are not regulated in feed and food globally. However, these and other POP-like substances accumulate in food of animal origin, potentially leading to food contamination, and should, therefore, be assessed. The magnitude of accumulation of some of these pollutants has been documented in research studies:

#### Brominated and brominated–chlorinated PBDD/Fs and PXDD/Fs

Brominated dioxins and furans (PBDD/Fs) have toxicities similar to those of PCDD/Fs and are, therefore, of concern [305, 306]. The UK food authority conducted a comprehensive survey on PCDD/Fs, PBDD/Fs and PXDD/Fs (mixed halogenated dioxins/furans) in British food, highlighting that up to 30% of TEQ could stem from PBDD/Fs, and an additional 20–50% from PXDD/Fs [307]. Eggs from free-range hens in Thailand and China have been found highly contaminated with PBDD/Fs [33]. PBDD/Fs are even formed from PBDEs during cooking of fish [308]. In Sweden, the PBDD/F-TEQ contribution to human background contamination reached up to 15% of TEQ [309], and firefighters in the USA had 20 times higher TEQ levels from PBDD/Fs compared to PCDD/Fs in their blood [310]. The major source of PBDD/Fs is aromatic brominated flame retardants, in particular PBDEs [311–313]. Based on measurements in e-waste plastic, it is estimated that the more than 1.3 million tonnes of PBDEs produced and used in consumer articles could have caused a total PBDD/F contamination in polymers in the order of 1000 t [312]. They are formed in the life cycle of PBDE-containing products, with highest releases at the end-of-life [314].

#### Chlorinated paraffins

Chlorinated paraffins (CPs) are the chlorinated semivolatile organic compounds with the highest production volume (1 million tonne/year), and were main substitutes for PCBs in open applications [315]. In May 2017, short-chain chlorinated paraffins (SCCPs) were listed as POPs under the Stockholm Convention, with a range of exempted uses [109]. For medium-chain chlorinated paraffins (MCCPs), a recent review also documented similar persistence and accumulation challenges [284]. Chlorinated paraffins bioaccumulate in meat/fat [316, 317] and might pose a risk for the future safe production of food from animal origin, considering their large use volumes in open applications and bioaccumulation potential. High levels of chlorinated paraffins (short-, medium- and long-chain chlorinated paraffins) have been detected in wildlife in China, accounting for more than 95% of all POPs detected in the biota [318, 319]. CPs in sewage sludge are in the mg/kg range [320, 321], and are currently transferred to agricultural soils via this pathway, accumulating and eventually contaminating food-producing animals and wildlife. SCCPs and MCCPs are also transferred from food contact materials such as baking ovens [322] or food blenders [323] to food and human intake.

#### Polybrominated flame retardants

In 2009, some brominated flame retardants were listed as POPs under the Stockholm Convention, including polybrominated diphenyl ethers (PBDEs) and polybrominated biphenyls (PBBs) and in 2013 hexabromocyclododecane (HBCD) was listed. The significance of contaminated soils and sediments to food contamination has been demonstrated for PBDEs [33, 324, 325]. Human exposure via eggs was shown to be a major pathway from e-waste recycling sites in China [324, 325]. In North America, where the largest amount of PBDEs has been used in the past [326], PBDE exposure of cattle/meat resulted from application of sewage sludge/biosolids [327]. For HBCD direct exposure of chicken and contamination of eggs have been documented [33, 328].

#### PFOS, PFOA and other per- and polyfluorinated alkylated substances (PFAS)

In 2009, the first fluorinated POPs, perfluorooctanesulfonic acid (PFOS) and related precursor chemicals, were listed under the Stockholm Convention. Widespread environmental pollution was discovered for PFOS, PFOA (perfluorooctanoic acid) and other PFAS. These POPs can bioaccumulate in meat [329]. The transfer and exposure of PFOS from soil to food animals have been demonstrated and reviewed showing that exposure from soil is a significant exposure pathway that needs to be considered in the assessment of human PFOS exposure [330]. Transfer factors from environmental contamination to food animals have been established for PFOS [330]. Assessments of a PFOS/PFAS-contaminated site impacting the groundwater of a farm have shown highest PFOS/PFAS levels in cattle, up to ppm level in blood [331]. Due to their water solubility, PFAS can accumulate in vegetables, fruits and grain, which can become an important exposure pathway for humans. Currently, more than 4000 PFAS are in use [332]. All are highly persistent or have highly persistent degradation products [333]. There are currently no limit values in soil or food. Only PFOS/PFOA and a few other PFAS have drinking water health advisories or limits (e.g., [334, 335]).

#### Other pollutants of concern

POPs are only one group of soil pollutants of concern [36]. FAO/ITPS considers the following other soil pollutants [35]:

- Inorganic compounds (e.g. heavy metals, metallic trace elements, metalloids and radionuclides).
- Other organic compounds (e.g. xenobiotic molecules, antibiotics, polycyclic aromatic hydrocarbons, mineral oil).
- So-called “chemicals of emerging concern” (CECs) in soil amendments (e.g., antibiotics in manure). CECs also include, for example, nanoparticles, pharmaceuticals and personal care products, estrogen-like compounds, antibiotics and hormones, flame retardants, detergents, currently used pesticides, plastics and microplastics, PFAS, various industrial chemical additives such as softeners, UV-stabilizers and antioxidants, and pollutants from open burning of electronic waste, such as chlorinated and brominated polycyclic aromatic hydrocarbons.
- Some organic wastes that can enhance the risk of spreading infectious diseases (e.g., untreated biosolids and wastewaters).

For a comprehensive and holistic protection of soils for sustainable food production, all these pollutants need to be monitored and controlled. One challenge is the impact of mixture toxicity, which is currently not considered in risk assessment. Furthermore, only approximately 100 chemicals are currently addressed by soil regulations, while approx. 140,000 chemicals are used in industrial applications and consumer products, with more than 500 of these chemicals having POP properties [336].

Bold approaches are needed to protect the soil and the environment from further pollution and degradation, such as the EU DG Environment strategy for a non-toxic environment, which is conducive to innovation and the development of sustainable substitutes, including non-chemical solutions [337, http://ec.europa.eu/environment/chemicals/non-toxic/index_en.htm].

### Management of pollutants and contaminated sites for safe food production

As mentioned in Sect. 2.7, there are a range of management measures for livestock farming, to reduce exposure on moderately contaminated areas. They have already been applied in a few cases, and have potential to be further developed and optimized.

#### Further compilation of practical experience regarding food contamination pathways

Experiences regarding PCDD/F and PCB exposure of food-producing animals have been compiled (Sect. 2.2; Figs. 1, 5). Further such experiences should be gathered and made public, to help mitigate exposure pathways, including for other POPs groups used in the technosphere (PFOS and other PFAS; PBDEs and other brominated flame retardants (BFRs); SCCPs) (for initial information see Sect. 3.5). These chemicals might have different exposure pathways due to different technical uses in products and different physico-chemical properties.

#### Guidance documents from international organisations and governments

To support safe food production and to avoid PCDD/F-, PCB- and other POP contamination of food of animal origin (meat, milk and eggs), international organisations and governmental bodies have developed some guidance documents.

The Food and Agriculture Organization (FAO) published the “Code of Practice for the Prevention and Reduction of Dioxin and Dioxin-like PCB Contamination in Food and Feeds” in 2006 [338].

Several documents were developed at the EU level, e.g., “Evaluation of the Occurrence of PCDD/PCDF and POPs in Wastes and Their Potential to Enter the Food Chain” [18] or “Guidelines for the enforcement of provisions on dioxins in the event non-compliance with the maximum levels for dioxins in food” [339].

Guidance documents have also been developed at a national level. For instance, the German Environmental Ministry has published a guidance on “Environmental protection—pillar for food safety to avoiding dioxin and PCB entry” [10]. The Chamber of Agriculture of the federal state of Lower Saxony developed leaflets for farmers on controlling PCDD/F and PCB input, and on cultivation on contaminated land [340, 341].

The recently developed national guidance documents consider more recent findings, which might be included in a possible update of the FAO Code of practice [338]. Some of the findings described in this review article, including the significance of soils, contaminated areas, and options for management measures and systematic screening could be considered in a possible update.

#### Cooperation between competent authorities

Often, specific information is managed by a certain ministry or regional authority. The topic of food contamination from the environment is a cross-cutting issue for the Ministry of Environment and the ministry responsible for food safety. To effectively address this topic, a close collaboration among ministries is needed. For instance, data on contaminated sites/contaminated water bodies are normally managed by the environmental ministry, whereas data on contamination of feed/food are normally handled by the ministry/authority responsible for food safety. These datasets need to be brought together to assess the impact of contaminated sites and waters on feed/food contamination. Furthermore, information on potentially contaminated sites could be used for developing monitoring strategies for food-producing animals. Competent authorities should cooperate and inform potentially affected people.

#### Inventory of POP-contaminated sites and updating soil standards to better consider livestock

Under the framework of the Stockholm Convention, inventory guidance documents for the newly listed POPs (PFOS, PBDEs, HBCD, PCNs, PCP and HCBD) have been developed, including a chapter on contaminated sites [154, 155, 342– 345]. Developing inventories of POP-contaminated sites, securing and, where feasible, remediating these sites can improve feed and food safety, while contributing to achieving several Sustainable Development Goals (see below) [346].

No soil standards have yet been developed for PFOS, PFOA, PBDEs or PBDD/Fs, even though human exposure via livestock has been demonstrated for these POPs (see above) [346]. To establish soil standards, the most critical exposure pathway (in terms of animals for food production) should be considered. For the reasons detailed above, this pathway is the consumption of meat/eggs from chickens that ingest polluted soil. Higher soil contamination limits might be established for less-exposed animals—although, again, particular care needs to be taken in relation to those animals whose liver or kidneys are consumed, such as pigs, beef and sheep.

Environmental Quality Standards (EQS) are concentrations of pollutants in water, sediment or biota that were derived to protect the aquatic environment and human health. EQS have been proposed by the EU Water Framework Directive and the Marine Framework Directive. The most sensitive protection goal for the derivation of EQS for dioxins and dioxin-like compounds was the protection of human health via consumption of fishery products. For those POPs lacking regulatory limits in food legislation, such EQS have been tailored according to toxicological guidance values for the consumption of fish and seafood in high consumers. This could possibly serve as model for other food and EQS for soils.

In addition to POP limits in soil, surface water and groundwater contamination are relevant for water-soluble POPs such as PFOS, PFOA and other PFAS. Limits for water are likely to be particularly important when groundwater or surface water is used for human or animal drinking water, for irrigation, or for aquaculture or fishing [346].

Soil pollution must be assessed in an integrated manner. POPs covered by the Stockholm Convention, which is ratified by 182 countries, could be a “vehicle” to initiate a more holistic soil pollution assessment, particularly in countries with developing and emerging economies often having no cadastre of polluted sites.

### Contribution towards the implementation of the sustainable development goals

As mentioned above, soil pollution has been highlighted by FAO as one of the ten major soil threats identified in the 2015 Status of the World’s Soil Resources report [35]. There is a direct link between the quality and safety of the food we eat and the level of soil pollution. Soil pollution has a direct impact on food security [338]. Therefore, soil protection and avoidance of further pollution and appropriate management of contaminated soils is needed for sustainable development. Several goals and indicators of the United Nations Sustainable Development Goals (SDGs) are of direct relevance to POPs pollution and other type of contaminated sites and soils [346]:

- Goal 2: End hunger, achieve food security and improved nutrition, and promote sustainable agriculture.
- Goal 3: Ensure healthy lives and promote wellbeing for all at all ages (specifically *Target 3.9: By 2030, substantially reduce the number of deaths and illnesses from hazardous chemicals and air, water and soil pollution and contamination*).
- Goal 6. Ensure availability and sustainable management of water and sanitation for all (specifically *Target 6.3: By 2030, improve water quality by reducing pollution, eliminating dumping and minimizing release of hazardous chemicals and materials, halving the proportion of untreated wastewater and substantially increasing recycling and safe reuse globally*).
- Goal 11: Make cities and human settlements inclusive, safe, resilient and sustainable.
- Goal 12: Ensure sustainable consumption and production patterns (specifically *Target 12.4: By 2020, achieve the environmentally sound management of chemicals and all wastes throughout their life cycle, in accordance with agreed international frameworks, and significantly reduce their release to air, water and soil in order to minimize their adverse impacts on human health and the environment*).
- Goal 15: Protect, restore and promote sustainable use of terrestrial ecosystems, sustainably manage forests, combat desertification, halt and reverse land degradation, and halt biodiversity loss (specifically *Target 15.1: By 2020, ensure the conservation, restoration and sustainable use of terrestrial and inland freshwater ecosystems and their services, in particular forests, wetlands, mountains and dry lands, in line with obligations under international agreements; and Target 15.3: By 2030, combat desertification, restore degraded land and soil*).

Soil pollution prevention by improved chemical and waste management and the management of contaminated soils need to be part of the activities for the implementation of these Sustainable Development Goals.

## Abbreviations

BAT: best available techniques
BBodSchV: Bundesbodenschutzverordnung (German Federal Soil Protection Ordinance)
BEP: best environmental practices
CNP: chloronitrophen
CPs: chlorinated paraffins
dl-PCBs: dioxin-like polychlorinated biphenyls
dm: dry mass, dry matter
EAF: electric arc furnace
EDC: ethylene dichloride
EEA: European Environment Agency
EFSA: European Food Safety Authority
EMEP: European Monitoring and Evaluation Programme
FAO: Food and Agriculture Organization of the United Nations
HBCD: hexabromocyclododecane
HCB: hexachlorobenzene
HCBD: hexachlorobutadiene
HCH: hexachlorocyclohexane
IPEN: International POPs Elimination Network
ITPS: Intergovernmental Technical Panel on Soils
MCCPs: medium-chain chlorinated paraffins
ndl-PCB: non-dioxin-like polychlorinated biphenyls
OCS: octachlorostyrene
PBDD/Fs: polybrominated dibenzo-*p*-dioxins and polybrominated dibenzofurans
PBDE: polybrominated diphenyl ethers
PBTs: persistent, bioaccumulative and toxic substances
PCBs: polychlorinated biphenyls
PCDD/Fs: polychlorinated dibenzo-*p*-dioxins and polychlorinated dibenzofurans
PCNs: polychlorinated naphthalenes
PCP: pentachlorophenol
PeCBz: pentachlorobenzene
PFOA: perfluorooctanoic acid
PFAS: per- and polyfluoroalkyl substances
PFOS: perfluorooctanesulfonic acid
POPs: persistent organic pollutants
PVC: polyvinyl chloride
PXDD/F: mixed halogenated dibenzo-*p*-dioxins and dibenzofurans
R&D: Research and Development
SCCPs: short-chain chlorinated paraffins
TDI: tolerable daily intake
TEF: toxicity equivalency factor
TEQ: toxic equivalent
TWI: tolerable weekly intake
UBA: Umweltbundesamt (German Environment Agency), German EPA
UNEP: United Nations Environment Programme
UNIDO: United Nations Industrial Development Organization
UPOPs: unintentionally formed POPs, unintentional POPs
WHO: World Health Organization
2,4-D: 2,4-dichlorophenoxyacetic acid
2,4,5-T: 2,4,5-trichlorophenoxyacetic acid

## Footnotes

1 Exposure and contamination of fish and other aquatic food-producing animals were addressed in the assessment study [34] and will be described in another paper, but are not addressed in this publication.

2 If an egg is above the EU regulatory limit, also the chicken meat is above the regulatory limit.

3 PCB contamination in sewage sludge decreased in the last decades [34, 107]. Today PCB-TEQ concentration is around 6 ng TEQ/kg dm in Switzerland and less in Spain and Germany [107] which has minor relevance to soil contamination.

4 Including chromated copper arsenate (CCA), one of most frequently used wood preservatives, which contaminates the ash with heavy metals [225].

5 The area-related application rates need to be taken into account which are up to six times higher than sewage sludge.

6 The PCB producing countries have elevated PCB levels in human milk [15], indicating a higher average use of PCB most likely in open applications leading to higher exposure.
